# An overview of nuclear medicine research in the UK and the landscape for clinical adoption

**DOI:** 10.1097/MNM.0000000000001461

**Published:** 2021-07-19

**Authors:** Jennifer D. Young, Maite Jauregui-Osoro, Wai-Lup Wong, Margaret S. Cooper, Gary Cook, Sally F. Barrington, Michelle T. Ma, Philip J. Blower, Eric O. Aboagye

**Affiliations:** a Department of Imaging Chemistry and Biology, School of Biomedical Engineering and Imaging Sciences, King’s College London; b National Cancer Imaging Translational Accelerator, Cancer Research UK; c Department of Surgery & Cancer, Division of Cancer, Imperial College London, London; d Department of Nuclear Medicine, Mount Vernon Cancer Centre, Mount Vernon Hospital, Northwood; e King’s College London and Guy’s and St Thomas’ PET Centre, King’s College London, King’s Health Partners, London, UK

**Keywords:** clinical studies, nuclear medicine commissioning, radiotracer production

## Abstract

Nuclear medicine contributes greatly to the clinical management of patients and experimental medicine. This report aims to (1) outline the current landscape of nuclear medicine research in the UK, including current facilities and recent or ongoing clinical studies and (2) provide information about the available pathways for clinical adoption and NHS funding (commissioning) of radiopharmaceuticals.

**Methods:**

Evidence was obtained through database searches for UK-based nuclear medicine clinical studies and by conducting a questionnaire-based survey of UK radiopharmaceutical production facilities. A recent history of clinical commissioning, either through recommendations from the National Institute for Health and Care Excellence (NICE) or through NHS specialised services commissioning, was compiled from publicly available documents and policies.

**Results:**

The collected data highlighted the UK’s active nuclear medicine research community and recent investment in new facilities and upgrades. All commissioning routes favour radiopharmaceuticals that have marketing authorisation and since 2017 there has been a requirement to demonstrate both clinical and cost-effectiveness. Whilst radiopharmaceuticals for molecular radiotherapy are well suited to these commissioning pathways, diagnostic radiotracers have not historically been assessed in this manner.

**Conclusions:**

We hope that by collating this information we will provide stimulus for future discussion and consensus statements around this topic.

## Abstract 2

Supplemental Digital Content is available in the text.

## Introduction

Nuclear medicine is a specialised area of clinical imaging and therapy that uses radioactive pharmaceuticals (radiopharmaceuticals) to diagnose, evaluate and treat diseases. It includes two diagnostic imaging modalities – gamma-scintigraphy (including single-photon emission computed tomography (SPECT)) and PET – and a treatment modality – targeted radionuclide therapy or molecular radiotherapy (MRT). Over 650 000 National Health Service (NHS) diagnostic imaging procedures utilised a diagnostic radiopharmaceutical (radiotracer) in the year 2019–2020 [1–3]. MRT is a smaller but rapidly expanding area of nuclear medicine (250% increase since 2007) with over 6000 procedures conducted in the UK in 2017 [4].

## Gamma-scintigraphy

Gamma-scintigraphy, including SPECT, utilises gamma-emitting radionuclides for diagnostic imaging of functional, physiological and metabolic processes. SPECT scans are often co-registered with anatomical CT scans. In the UK, the most common gamma-scintigraphy scans include bone, lung, myocardium and kidney scans [5]. Over 85% of procedures utilise radiotracers incorporating technetium-99m, which emits 140 keV gamma photons [5]. This has a 6-h half-life, so ^99m^Tc-labelled radiotracers must be produced on the day of administration, typically at hospital-based radiopharmacy production units (RPUs), from nonradioactive starting materials (radiopharmaceutical kits) and a source of technetium-99m (molybdenum-99/technetium-99m generator), using aseptic techniques.

## PET

PET is a diagnostic imaging technique that uses positron-emitting radionuclides and locates the radiotracer using the two 511 keV photons emitted in opposite directions when positrons and electrons mutually annihilate. PET data are typically co-registered with anatomical imaging on PET-CT, PET-MRI or more recently total-body PET-CT scanners [6,7]. The most commonly used PET radionuclide, fluorine-18, has a half-life of 109.8 min and is produced in a compact particle accelerator – a cyclotron. It can then be combined with nonradioactive chemical precursors to yield the desired ^18^F-labelled radiotracer, which is administered on the day of manufacture. The most common PET radiotracer is [^18^F]Fluorodeoxyglucose ([^18^F]FDG), a radioactive glucose analogue used for imaging a wide range of cancers, myocardial viability, vasculitis, infection and neurological disorders [8]. [^18^F]FDG accounts for over 90% of clinical PET-CT scans in England [9].

## Molecular radiotherapy

MRT utilises alpha- or beta-emitting radionuclides, typically with half-lives in the order of days that have a therapeutic effect when accumulated at disease sites. The most common form of MRT in the UK is [^131^I]NaI (sodium iodide), used to treat benign thyroid disease and thyroid cancer [4,10]. MRT is also well-established for treating bone metastasis and related bone pain palliation [11]. The key radiopharmaceuticals for this indication are beta-emitting strontium-89 dichloride (Metastron) and samarium-153 lexidronam (Quadramet) and alpha-emitting radium-223 dichloride (Xofigo).

## Aims

This report aims to (1) outline the current landscape of UK nuclear medicine research, including facilities and recent clinical studies and (2) describe available pathways for clinical adoption and NHS commissioning of radiotracers.

## UK clinical study landscape

Nuclear medicine clinical studies span from first-in-human studies in healthy volunteers to randomised controlled trials (RCTs) with patients followed up over years. Studies can focus on establishing the safety, dosimetry and efficacy of a radiopharmaceutical for imaging or MRT or answering basic science and experimental medicine questions. Studies can be observational – where the investigator does not intervene but records what happens to those enrolled – or interventional – where the investigator intervenes in a defined way and records the outcome. The intervention may be use of a novel radiotracer or MRT, or another treatment, such as a pharmaceutical drug or surgery. The radiotracer itself can be the focus of the investigation or a tool used for patient screening, probing biological processes or monitoring treatment response.

**Fig. 1:**
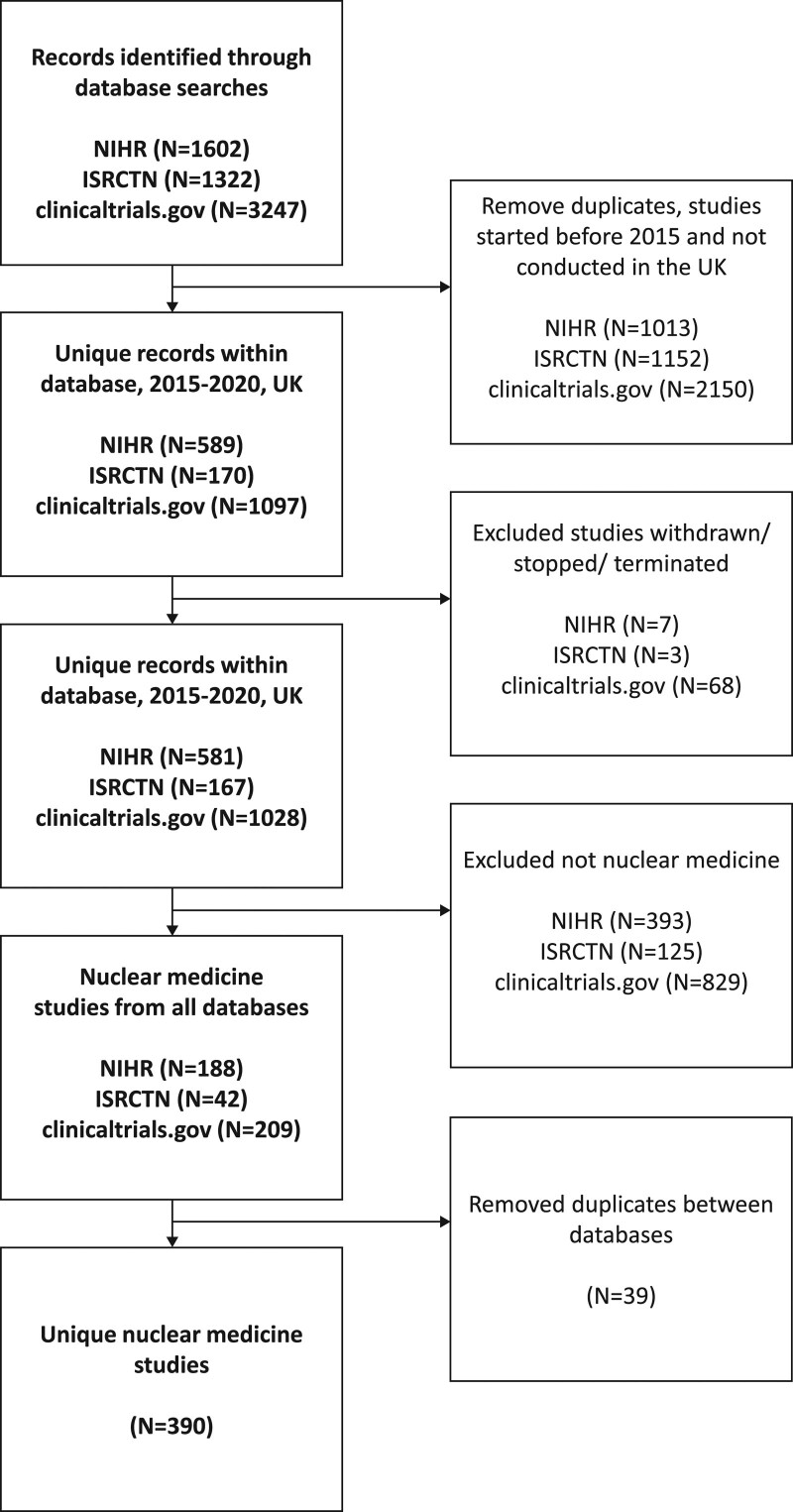
A flow diagram demonstrating the process used to sort the original database outputs to obtain a final list of unique nuclear medicine studies.

We conducted a search in November 2020 of the National Institute for Health Research Clinical Research Network (NIHR CRN) portfolio, the ClinicalTrials.gov database and the ISRCTN registry (all free access databases), for UK nuclear medicine studies starting between 2015 and 2020 (inclusive), using the search terms in Table 1. Results, sorted as described in Fig. 1, included all studies in which a radiotracer was used, including studies where the research focus was on the radiopharmaceutical and studies where the radiopharmaceutical was used only for screening or monitoring. Further data, such as the radiopharmaceutical used (which in some cases was inferred from how it was used; e.g. [^18^F]FDG was the presumed radiotracer when PET-CT was used to stage tumours) and whether the study was a clinical trial of an investigational medicinal product (CT(IMP)), were extracted manually from each study record to ensure equivalent data were available for each study regardless of the database it originated from.

**Table 1:** Search terms used in each of the three clinical study databases (NIHR CRN portfolio, the ClinicalTrials.gov database and the ISRCTN registry) to identify all nuclear medicine studies and the information extracted from each. It was not possible to distinguish SPECT from gamma-scintigraphy in many of the records therefore these were combined into one service group

Search terms used	‘nuclear’; ‘radionuclide’; ‘dosimetry’; ‘SPECT’; ‘gamma’; ‘PET’; ‘Positron’; ‘scintigraphy’; ‘radiotracer’; ‘227Th’; ‘thorium’; ‘223Ra’; ‘radium’; ‘188Re’; ‘rhenium’; ‘90Y’; ‘yttrium’; ‘89Zr’; ‘zirconium’; ‘68Ga’; ‘gallium’; ‘177Lu’; ‘lutetium’; ‘99mTc’; ‘technetium’; ‘11C’ ‘carbon’; ‘18F’ ‘fluorine’; ‘FDG’ ‘64Cu’; ‘copper’; ‘123I’; ‘131I’; ‘iodine’.	
Feature	Categories	Notes
Reference	NIHR XXXXX	Defined in database
	ISRCTNXXXXXXXX	
	NCTXXXXXXXX	
Study Name	Free format name	Defined in database
Service	SPECT or gamma-scintigraphy; PET; MRT	Defined by authors
Tracer	Free format name of radiotracer manually identified from study record.	Defined by authors
Study type (interventional/ observational)	Interventional; observational	Defined in databases
Study phase	Phase 1; phase 2; phase 3; phase 4; NA	Defined in databases. If defined as phase1/phase 2 was categorised for analysis as phase 2.
Disease category/ speciality	Cancer, neurology, cardiovascular; other	Defined by author
Industry funded	Industry; other	Defined by author
CT(IMP)/non-CT(IMP)	CT(IMP)/non-CT(IMP)	Defined by author
		If interventional and phase 1-4 from database - CT(IMP)
		If interventional NA phase - non-CT(IMP).
IMP/Non-IMP	IMP; non-IMP; observational; interventional no phase (non-CT(IMP))	Defined by authors.
		If names as an investigational drug for the trial - IMP.
		If not – non-IMP
		Categorisation was also reviewed against the MHRA’s algorithm

All interventional studies categorised as phase 1–4 trials were inferred to be CT(IMP)s and reviewed against the Medicines and Healthcare products Regulatory Agency (MHRA) algorithm ‘Is it a clinical trial of a medicinal product?’ [12] to classify the radiotracers either as investigational medicinal products (IMP) or non-IMPs. Studies were broadly grouped into specialities: cancer, neurology, cardiovascular and other, and categorised as industry-funded if at least one funder was a commercial entity. The information obtained from each study record is summarised in Table 1. All included studies are listed in Supplementary Table 2, Supplemental digital content 1, http://links.lww.com/NMC/A199. We identified 390 distinct nuclear medicine studies initiated in the UK between 2015 and 2020. These were at different stages of completion, so details may change and some results are not yet available.

**Fig. 2:**
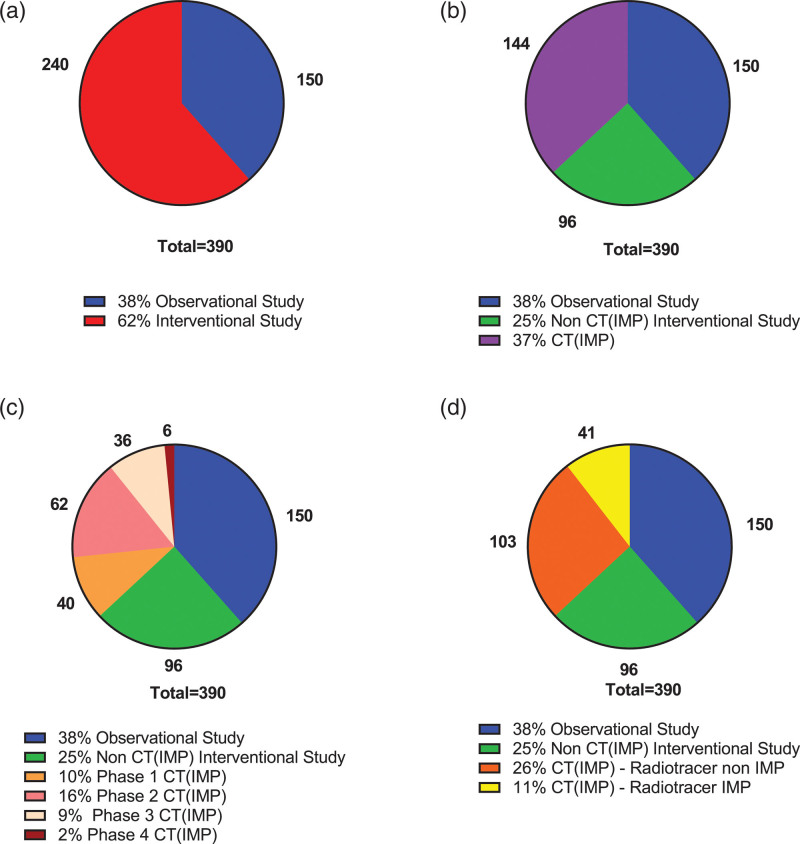
(a) Proportion of interventional and observational studies. (b) Same information as (a) but the interventional studies are split into clinical trials involving an investigational medicinal product (i.e. CT(IMP)) and interventional studies that do not involve an investigational medicinal product (i.e. non-CT(IMP)). (c) CT(IMP) studies are split into the different trial phases (phase 1–4). (d) The proportion of CT(IMP) studies where the IMP is a radiotracer.

**Fig. 3:**
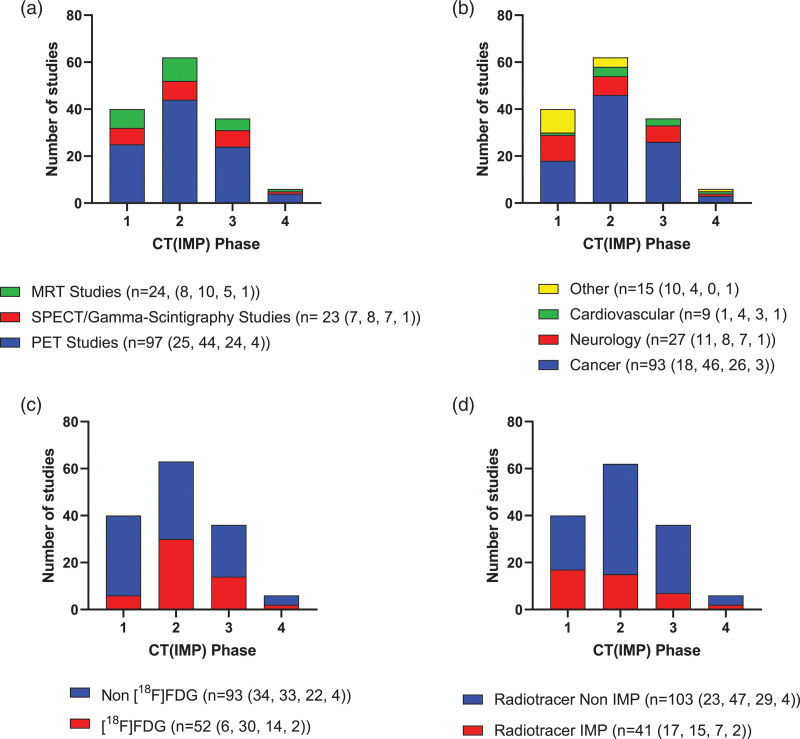
Clinical trials involving an investigational medicinal product (CT(IMP)s) split into phase 1–4 trials. (a) Contribution of each nuclear medicine service area to the number of CT(IMP)s of each phase. (b) Research speciality of the studies within each phase. (c) Proportion of studies within each phase that utilise the radiotracer [^18^F]FDG – the most commonly used PET radiotracer. (d) Proportion of CT(IMP)s from each phase where the IMP is a radiotracer.

PET studies were the most frequent (*n* = 309), followed by gamma-scintigraphy/SPECT (*n* = 52) and MRT (*n* = 29). Cancer, neurology and cardiovascular research represented 46, 26 and 16% of studies, respectively. Cancer accounted for 40% of PET studies, 46% of SPECT/gamma-scintigraphy and 100% of MRT studies. Within PET, there were many neurology (*n* = 95) and cardiovascular (*n* = 60) studies, while in gamma-scintigraphy/SPECT, only three cardiovascular and five neurology studies were identified. Industry-funded projects accounted for 38% of studies, varying by modality and disease type; 55% used PET to investigate either cancer or neurology. Most studies were interventional rather than observational. In only 17% (*n* = 41) of interventional studies was the radiopharmaceutical an IMP (Figs. 2, 3).

Ninety-three radiopharmaceuticals featured in the survey – 17 gamma-scintigraphy/SPECT radiotracers, 61 PET radiotracers and 15 MRT agents (Supplementary Table 3, Supplemental digital content 1, http://links.lww.com/NMC/A199) – spanning from radiopharmaceuticals common in clinical practice, such as [^18^F]FDG, to those being investigated in first-in-human studies, such as [^18^F]BF_4_ (NIHR19094), [^18^F]FPIA (NIHR35889) and [^227^Th]Th-BAY2287411 (NCT03507452). The most common PET radiotracers were [^18^F]FDG (*n* = 116), [^18^F]NaF (*n* = 29) and ‘amyloid PET’ radiotracers (*n* = 24), accounting for 38, 9 and 8% of PET studies, respectively. Thirty-four (11%) PET studies involved a radiotracer unique to that study. The most utilised gamma-scintigraphy/SPECT technique (36%) was a bone scan, where the radiotracer was surmised to be [^99m^Tc]Tc-MDP, [^99m^Tc]Tc-HDP or [^99m^Tc]Tc-DPD. In MRT, ten studies used [^223^Ra]RaCl_2_, five used [^131^I]NaI and all remaining studies used a radiopharmaceutical unique to that study.

## UK radiotracer production landscape

The volume and reliability of production and supply of radiotracers affects the range of procedures used. An additional constraint for many diagnostic radiotracers is that their short half-lives impose production at the same site and on the same day as administration to patients.

As medicinal products, radiopharmaceuticals must be made according to UK Human Medicines Regulations 2012 [13]. Here we describe marketing authorisation (MA) and manufacturer licences that can be issued to UK facilities producing radiopharmaceuticals.

## Marketing authorisation

MA (formerly called a product licence [14,15]) is required before a medicinal product can be marketed (sold, supplied or exported) in the UK. MA for medicines meeting standards set by a medicines regulator is granted to marketing authorisation holders (MAH) [16], which are almost exclusively pharmaceutical companies.

## Manufacturer licences

Three types of manufacturer licences can be issued for UK radiotracer production: (1) Manufacturing and Importation Authorisation (MIA) to manufacture medicinal products granted MA; (2) ‘Specials’ licence (MS), to manufacture medicinal products without MA as ‘specials’; (3) licence to manufacture investigational medicinal products (MIA(IMP)), for investigation within a clinical trial. All three are granted by the MHRA, which inspects sites regularly to ensure compliance with Good Manufacturing Practice (GMP) regulations and other licence conditions. For MIA licences, inspections are product-specific. Radiopharmaceuticals produced under a MIA licence are labelled with the MA number and can be marketed by the MAH in specified countries. For sites holding only MS licences, inspections are site-specific; products manufactured under this licence cannot be labelled as GMP. The basis of inspections of sites holding MIA(IMP) licences is defined in the EU Clinical Trials Regulation EU No 536/2014; details about the manufacture of IMPs to GMP standards can be found in Annex 13 of 2001/83/EC and in Supplementary Table 4, Supplemental digital content 1, http://links.lww.com/NMC/A199.

## Nonlicensed activities

Section 10 of the UK Medicines Act 1968, incorporated into the Human Medicines Regulations 2012, describes professional exemptions for pharmacists [13]. These allow, in certain circumstances, the preparation of pharmaceuticals to be undertaken without a manufacturer licence [17]. Radiopharmacies can operate under Section 10 if activities are supervised by a pharmacist and limited to preparation of radiopharmaceuticals in closed systems from sterile medicinal products with MA (i.e. nonradioactive radiopharmaceutical kits and radionuclide generator eluates). Additionally, products must have an expiry date of less than one week from preparation. These facilities must meet GMP standards but are audited by an approved pharmacy regional quality assurance specialist rather than the MHRA, as no licensed activities are conducted [18].

## Survey of radiotracer manufacturing facilities

A questionnaire about radiotracer production facilities was sent to 31 NHS and university radiopharmaceutical production facilities, and a modified version to five commercial entities (Supplementary Figures 3 and 4, Supplemental digital content 1, http://links.lww.com/NMC/A199). Responses were received between April 2020 and January 2021 from 11 noncommercial academic and NHS-run radiochemistry facilities that use cyclotron-produced radionuclides (carbon-11 and fluorine-18) to manufacture radiotracers for human use (Supplementary Table 5, Supplemental digital content 1, http://links.lww.com/NMC/A199). Of these facilities, those currently holding a manufacturing licence and their reported radiotracers for human use are listed in Table 2. Five of them have an on-site cyclotron, and one relies on an external source of fluorine-18. All the cyclotrons can produce fluorine-18 and carbon-11 and some produce a wider range of radionuclides (Table 2). Nevertheless, the only cyclotron-produced radionuclides currently being incorporated into radiotracers for human use are fluorine-18 and carbon-11. Table 2 shows that the range of radiotracers produced varies widely across centres. While [^18^F]FDG is produced at four sites, each of the other radiotracers is produced at only one or two; just one reported producing ^11^C-radiotracers.

**Table 2:** Summary of the six noncommercial radiochemistry facilities in the UK that produce radiotracers for human use from radionuclides produced in a cyclotron. This includes their cyclotron capabilities and the radiotracers they can currently produce for human use under their manufacturer ‘specials’ licence or manufacturer licence for investigational medicinal products

	Cyclotron	Radionuclide capabilities	^18^F-labelled radiotracers	^11^C-labelled radiotracers	MHRA Licences
Centre for Radiopharmaceutical Chemistry	No	NA	[^18^F]MK6240	–	MS
			[^18^F]DPA-714		MIA(IMP)
University College London			[^18^F]FDOPA		
			[^18^F]D4- Fluoro –choline		
			[^18^F]FPIA		
			[^18^F]NAV4694		
Positron Emitting Radiopharmaceutical Laboratory	Yes	Fluorine-18	[^18^F]FDG	–	MS
		Carbon-11			MIA(IMP)
King’s College London		Oxygen-15			
		Nitrogen-13			
		Copper-64			
Wolfson Brain Imaging Centre	Yes	Fluorine-18	[^18^F]FLT	[^11^C]Acetate	MS
		Carbon-11	[^18^F]FMISO	[^11^C]Methionine	MIA(IMP)
University of Cambridge		Oxygen-15	[^18^F]GE-226	[^11^C]Metomidate	
		Copper-64	[^18^F]AV-1451	[^11^C]Leucine	
			[^18^F]CETO	[^11^C]PIB	
			[^18^F]FDOPA	[^11^C]PK11195	
				[^11^C]UCB-J	
Wales Research and Diagnostic PET Imaging Centre	Yes	Fluorine-18	[^18^F]FDG	–	MS
		Carbon-11	[^18^F]F-PSMA-1007		
Cardiff University		Oxygen-15	[^18^F]Fallypride		
		Nitrogen-13	[^18^F]FDOPA		
		Gallium-68	[^18^F]Flumazenil		
		Zirconium-89			
		Vanadium-48			
Edinburgh Imaging	Yes	Fluorine-18	[^18^F]FDG	–	MS
		Carbon-11	[^18^F]Fluoro-choline		MIA(IMP)
University of Edinburgh		Oxygen-15	[^18^F]NaF		
			[^18^F]GP1		
			[^18^F]Flutemetamol		
West of Scotland PET Centre	Yes	Fluorine-18	[^18^F]FDG	–	MS
		Carbon-11	[^18^F]FMISO		MIA(IMP)
NHS Greater Glasgow and Clyde					

The Molecular Imaging Research Centre in Hull and the PET Tracer Production Unit in Newcastle are new facilities, planning to obtain their MS licence and start production in 2021. The PROx PET radiochemistry facilities at Oxford have been completed, but when licensing and production will commence is unknown. Several sites are undergoing refurbishment and upgrades: the John Mallard Scottish PET Centre, NHS Grampian, the University of Aberdeen, where a cyclotron and new hot-cells are being installed; the Positron Emitting Radiopharmaceutical Laboratory, King’s College London, where a new cyclotron, hot-cells and isolators have been installed; and Edinburgh Imaging, University of Edinburgh, where additional hot-cells and dispensers will be installed. This shows a continued investment in PET and cyclotron facilities, but restricts current capacity for clinical supply and research studies at these sites.

In contrast, the Wolfson Molecular Imaging Centre, the University of Manchester, which had a cyclotron and MS and MIA(IMP) licences enabling radiotracer provision for academic and industry-led projects, closed in 2020. Consequently, radiotracers produced solely by this site are no longer available in the UK (Supplementary Table 8, Supplemental digital content 1, http://links.lww.com/NMC/A199).

We received 14 responses from noncommercial sites producing radiotracers from noncyclotron-produced radionuclides (generator- or reactor-produced); all RPUs are based within NHS hospitals (Supplementary Table 6, Supplemental digital content 1, http://links.lww.com/NMC/A199). All 14 RPUs reported manufacturing technetium-99m radiopharmaceuticals using molybdenum-99/technetium-99m generators and radiopharmaceutical kits with MA (Supplementary Table 9, Supplemental digital content 1, http://links.lww.com/NMC/A199). Some also produce [^111^In]In-DTPA-octreotide and three sites produce other^99m^Tc-labelled and ^111^In-labelled radiotracers as specials or IMPs. Gamma-emitting radiotracers with MA received from commercial suppliers ready for administration were also reported (Supplementary Table 10, Supplemental digital content 1, http://links.lww.com/NMC/A199).

Germanium-68/gallium-68 generators and the production of ^68^Ga-labelled radiopharmaceuticals for PET imaging have been implemented at seven UK RPUs. All produce ^68^Ga-labelled somatostatin analogues ([^68^Ga]Ga-SSR) to image neuroendocrine tumours. Five also produce ^68^Ga-labelled radiotracers for prostate-specific membrane antigen-expressing tumours ([^68^Ga]Ga-PSMA) for imaging prostate cancer (Table 3). Only one produces other ^68^Ga-labelled radiotracers for clinical trials.

**Table 3:** Summary of the radiopharmacy production units that reported producing ^68^Ga-labelled radiotracers or radiopharmaceuticals for molecular radiotherapy and the manufacturer licences they hold

RPU	[^68^Ga]Ga-SSR	[^68^Ga]Ga-PSMA	MRT	MHRA licence
Radiopharmacy Unit, Barts Health NHS Trust	[^68^Ga]Ga-DOTA-TATE	[^68^Ga]Ga-PSMA-11	–	MS and MIA(IMP)
	NETSPOT kit	ANMI kit		
Radiopharmacy Department, Queen Elizabeth Hospital, Birmingham	[^68^Ga]Ga-DOTA-TOC	–	–	MS
	SOMAkit TOC kit			
Radiopharmacy, Nuclear Medicine, Addenbrookes Hospital, Cambridge University Hospitals NHS Foundation Trust	[^68^Ga]Ga-DOTA-TATE	[^68^Ga]Ga-PSMA-11	–	MS
	NETSPOT kit	ANMI kit		
Guy’s Radiopharmacy, Guy’s & St Thomas’ Foundation NHS Trust	[^68^Ga]-HA-DOTA-TATE	[^68^Ga]Ga-PSMA-11	–	MS and MIA(IMP)
	Automated synthesis unit	ANMI kit		
	[^68^Ga]Ga-DOTA-TATE	[^68^Ga]Ga-THP-PSMA		
	NETSPOT kit	GalliProst kit		
	[^68^Ga]Ga-DOTA-TOC			
	SOMAkit TOC kit			
Radiopharmacy Unit, The Christie NHS Foundation Trust	[^68^Ga]Ga-DOTA-TOC			
	SOMAkit TOC			
	(previously [^68^Ga]Ga-DOTA-NOC using automated synthesis unit)	–	–	MS
Radiopharmacy, Nuclear Medicine, Royal Free London NHS Foundation Trust	[^68^Ga]Ga-DOTA-TATE	[^68^Ga]Ga-PSMA-11	[^90^Y]Y-anti-CD66	MS and MIA(IMP)
	Automated synthesis unit	ANMI kit		
	[^68^Ga]Ga-DOTA-TATE			
	NETSPOT kit			
Radiopharmacy Department, The Royal Marsden NHS Foundation Trust	[^68^Ga]Ga-DOTA-TATE	[^68^Ga]Ga-PSMA-11	[^177^Lu]Lu-PSMA	MS
	NETSPOT kit	ANMI kit	[^90^Y]Y-DOTA-TATE	
	[^68^Ga]Ga-DOTA-TOC			
	SOMAkit TOC kit			
Radiopharmacy Department, University Hospitals Southampton NHS Foundation Trust	–	–	[^177^Lu]Lu-PSMA (antibody)	MS and MIA(IMP)
			[^227^Th]Th-PSMA (antibody)	
			[^227^Th]Th-mesothelium	
			[^90^Y]Y-anti-CD66	

In MRT, most radiopharmaceuticals with MA are delivered ready for administration and not manufactured at the hospital site. However, some sites produce lutetium-177, thorium-227 and yttrium-90 MRT radiopharmaceuticals as specials or IMPs (Table 3). The UK cannot produce these radionuclides; they are shipped from mainland Europe.

Four commercial suppliers responded: Alliance Medical Radiopharmacy Ltd, Advanced Accelerator Applications (AAA), Siemens Healthcare (Healthineers, PETNET UK) and Curium Pharma UK. Alliance Medical and Siemens Healthcare have multiple UK production sites with a cyclotron and focus on producing ^18^F-labelled radiotracers, mainly [^18^F]FDG. Both hold MIA and MS licences and UK MA for [^18^F]FDG – marketed as Fludeoxyglucose (^18^F) Injection by Alliance Medical and as Metatrace by Siemens Healthcare. Both produce [^18^F]NaF and [^18^F]Fluorocholine as ‘specials’. Additionally, Siemens Healthcare produces [^18^F]F-PSMA-1007 as a ‘special’ and Alliance Medical is a named manufacturer on the MA for [^18^F]Florbetaben (Neuraceq) held by Life Radiopharma Berlin GmbH. AAA have no UK manufacturing facilities, but supply DOPAVIEW (6-[^18^F]Fluoro-L-dihydroxyphenylalanine, for which they hold MA) manufactured in France to the UK. AAA supply generators and kits for producing [^68^Ga]Ga-SSR and the MRT Lutathera, which also targets somatostatin receptors. Curium Pharma import and supply radiopharmaceutical kits, generators and radiopharmaceuticals produced at their facilities in the Netherlands and France. They also operate a dispensing radiopharmacy in London, which holds a MS licence and supplies ^99m^Tc- and ^68^Ga-labelled radiopharmaceuticals as ‘specials’.

The company Invicro also forms part of the UK’s research radiotracer production landscape, providing translational radiochemistry services and manufacturing novel radiotracers for clinical studies within their London clinical imaging facilities (including ^11^C and ^18^F-labelled radiotracers) [19].

## UK commissioning landscape

To develop radiotracers for NHS adoption, the research community needs to be aware of the routes to routine service use and recent changes to them, which we outline below based on publicly-available National Institute for Health and Care Excellence (NICE) and NHS policies and supporting documents. There are two main routes for approval and adoption:

- Recommendation by NICE through published guidance or guidelines (only available to medicinal products with UK MA or CE marked medical devices).
- Direct commissioning through specification in an NHS Specialised Service Commissioning policy (open to unlicensed medicinal products, although those granted MA are preferred; but only open to services that are nationally commissioned, including PET-CT but no other nuclear medicine interventions).

Thus, the most likely first step towards NHS adoption of a radiopharmaceutical is obtaining MA. Therefore, we first discuss evidence required to obtain MA. The application fee for a UK National MA in 2021–2022 is £92 753 [20].

## Marketing authorisation

Article 8 of Directive 2001/83/EC specifies the compulsory documents for MA applications, which include results of the following in relation to the medicinal product and its constituent active substances [21].

- Pharmaceutical tests
- Preclinical tests
- Clinical trials

The clinical trial data included depend on the specifics of the medicinal product and the type of MA sought, and typically include phase 1, phase 2 and phase 3 trials. To provide relevant examples for nuclear medicine, here we describe evidence used to support MAs for Axumin ([^18^F]Fluciclovine) and Lutathera ([^177^Lu]Lu-oxodotreotide). Further details are provided in Supplementary Tables 15 and 16, Supplemental digital content 1, http://links.lww.com/NMC/A199. Blue Earth Diagnostics Ltd received MA for Axumin, indicated for recurrent prostate cancer, in 2017 (EMEA/H/C/004197). AAA was granted MA for Lutathera, indicated for gastroenteropancreatic neuroendocrine tumors (GEP NETs), in 2017 (EU/1/17/1226/001). These two applications differ in ways that affect the data supplied for MA: (1) Axumin is a PET-CT radiotracer whereas Lutathera is an MRT; (2) Axumin is indicated for a high incidence cancer – prostate – whereas Lutathera has orphan designation (GEP NETs are rare). Nine trials were cited for Axumin and two for Lutathera. However, their designs and endpoints differed: Axumin trials focused on diagnostic performance rather than patient outcome and were not RCTs, whereas the Lutathera phase 3 trial was a RCT with progression-free survival as a primary endpoint (patients were followed up for 5 years but follow-up data were not available when MA was granted).

## Health technology assessment

In common with most third-party payers, the NHS specifies processes to assess new health technology. NICE produces guidance, guidelines and advice to assist or mandate the adoption of products and practices across the NHS. These different publication types have different implications for NHS adoption and funding; see Supplementary Table 14, Supplemental digital content 1, http://links.lww.com/NMC/A199. NICE evaluation programs for health technologies (typically medicinal products granted MA) review clinical- and cost-effectiveness of treatments and include (1) technology appraisal guidance for new treatments; (2) highly specialised technology guidance for specialised treatments for very rare (ultraorphan) conditions. The NHS is legally obliged to fund technologies recommended through these types of guidance.

Evaluation programs for NHS adoption of medical technologies (typically devices, diagnostics or digital technologies granted CE marks) include (1) medical technologies guidance for new medical devices; (2) diagnostics guidance for new diagnostic technologies. The NHS is not obliged to fund the recommendations made by NICE through these types of guidance.

Below we examine how exemplar radiopharmaceuticals discussed in the MA section were reviewed by NICE.

## Exemplar 1 – Lutathera – NICE technology appraisal

Results of the same two clinical trials used to support MA were provided as evidence for the technology appraisal [22] (Supplementary Table 16, Supplemental digital content 1, http://links.lww.com/NMC/A199). The NICE guidance established that there was an unmet need for treatment for metastatic NETs and identified three comparators in current NHS practice: (1) octreotide (2) everolimus and (3) sunitinib; with octreotide defined as best supportive care [22]. The committee then reviewed the trial evidence to establish clinical effectiveness against the comparators and concluded that Lutathera was more effective than current treatment for GEP NETs. Cost-effectiveness was assessed using incremental cost-effectiveness ratios. For Lutathera, the cost was in the range NICE considers cost-effective – below £30 000 per quality-adjusted life-year (QALY) gained [14,23] – for both pancreatic and gastrointestinal NETs [22].

During a NICE technology appraisal, if a companion diagnostic is required to assess eligibility for treatment, the cost-effectiveness assessment considers both that companion diagnostic and the therapy [23,24]. For Lutathera, the scans required to determine somatostatin receptor expression in a tumour prior to therapy were already part of NHS standard of care (e.g. [^111^In]In-octreotide, [^99m^Tc]Tc-octreotide or [^68^Ga]Ga-SSR), therefore these were not included in the costing model [22].

After establishing both clinical and cost-effectiveness, the committee recommended Lutathera as a cost-effective use of NHS resources for treating somatostatin receptor-positive GEP NETs in people with progressive disease.

## Exemplar 2 -Axumin - NICE Medtech innovation briefing

No examples exist of novel PET radiotracers undergoing NICE technology appraisal into guidance [25]. However, in 2019, NICE published advice on Axumin [26] in the form of a Medtech Innovation Briefing, in which NICE reviewed the technology, its role in the treatment pathway, the published evidence and likely costs. New clinical evidence was included that was not available for the MA application: one meta-analysis, two RCTs and three nonrandomised observational studies (Supplementary Table 18, Supplemental digital content 1, http://links.lww.com/NMC/A199). It concluded that Axumin PET/CT can detect local and distant prostate cancer recurrences across a wide range of prostate-specific antigen levels. However, several concerns were highlighted, which we examine here, as they raise issues relevant to all PET imaging agents.

(1) Evidence that patient outcomes were improved by a PET-CT scan was poor. Although it was demonstrated that Axumin scans changed patient management compared with standard imaging, only weak evidence linked this change to improved outcomes. It was concluded that ‘longer-term studies or patient registries would be needed to assess the effect of Axumin-guided therapy on clinical outcomes such as earlier detection of recurrence, subsequent treatment and progression-free survival’. PET-CT studies commonly look at management changes, but rarely at changes in clinical outcome, as these rely on management changes due to the diagnostic test and ongoing management decisions.

(2) Comparator and population selection did not reflect UK practice. Only one of the six evidential studies was conducted in the UK (NCT02578940); the others were deemed irrelevant to NHS practice.

(3) A wide population of patients was eligible for scanning. The report stated: ‘understanding the performance of Axumin PET/CT in different populations, such as in people grouped by previous treatment, Gleason score and PSA doubling times, may provide information that could help inform the selection of patients for scanning’. By narrowing the eligible population, cost and clinical effectiveness of an intervention can often be improved, by increasing the proportion of patients moved from the wrong treatment pathway to the correct one.

## National commissioning policies for PET-CT

In NHS England, NHS Scotland and NHS Wales PET-CT is centrally commissioned, unlike other imaging modalities. An overview of each nation’s process follows. Indications for which radiotracers are commissioned in each nation are listed in Supplementary Tables 19–21, Supplemental digital content 1, http://links.lww.com/NMC/A199.

NHS Scotland: The Scottish Clinical Imaging Network (SCIN) was commissioned in 2014 by NHS Scotland [27]. In 2015, the Scottish Government Health and Social Care Directorate Diagnostic Steering Group instigated a SCIN PET-CT Working Group with a remit to develop evidence-based PET-CT guidelines [27]. Published in 2017 [28], these were based on the 2016 Intercollegiate Standing Committee on Nuclear Medicine (ICSCNM) report: Evidence-based indications for the use of PET-CT in the UK [8]; they have been updated regularly, including six guidelines introduced or updated in Nov 2020 [29–34].

NHS Wales: PET-CT in Wales is commissioned by the Welsh Health Specialised Services Committee. The commissioning policy, CP50a PET, was last updated in August 2020 [3] based on the 2016 ICSCNM report [8].

NHS England: Since 2013, when the Health and Social Care Act 2012 [35] came into force, NHS England has been responsible for commissioning 146 specialised services, of which PET-CT services is one [36]. This is unique among imaging modalities; all others, including wider nuclear medicine activities, are commissioned by regional Clinical Commissioning Groups following NICE guidelines and guidance. The current NHS England Clinical Commissioning Policy Statement for PET-CT (2015) [37] defines radiotracers and indications for which PET-CT services are commissioned and includes all indications recommended in the 2013 ICSNM guidelines [38].

In 2017, NHS England implemented a new commissioning process for specialised services [39], described in Supplementary Table 23, Supplemental digital content 1, http://links.lww.com/NMC/A199. In short, the Specialised Services Commissioning Committee assesses the clinical benefit and cost of new proposals and all 146 specialised service propositions requiring additional funding are ranked based on incremental clinical benefit and incremental cost. Only those providing the best value for money, whether therapeutic or diagnostic, are funded within the available budget. This new process requires stronger evidence of clinical effectiveness than the 2015 PET-CT Commissioning Policy Statement. No PET-CT Commissioning Policy Statements have been published since 2015 and three PET-CT proposals have been rejected [40]: Axumin, [^68^Ga]Ga-PSMA and an amyloid radiotracer. Reasons for rejection were not published, although detailed feedback was provided to applicants.

## Discussion

Radiopharmaceuticals are required for managing patient pathways and therapy, and for experimental medicine. Regulation, commissioning and reimbursement are country-specific processes likely to influence commercial investment in radiotracer development and clinical trials. We hope that the information collated in this report will stimulate discussion and consensus statements around this topic.

### Clinical study landscape in the UK

The collected data highlight the UK’s active research community but show that while radiopharmaceuticals were IMPs in most MRT studies only a small proportion of the diagnostic studies conducted were CT(IMP)s with the radiotracer as an IMP – a prerequisite for MA and the highest quality evidence for commissioning. Although this report focuses on research to support clinical adoption, the database search results were not limited to studies with this purpose, a significant proportion were the studies pursuing various scientific and experimental medicine questions and use of established diagnostic radiotracers to measure the performance of new treatments. Therefore, although many radiotracers are involved in clinical studies, it is unlikely many of these will be commissioned.

### UK radiotracer production landscape

The survey data provide an overview of radiopharmaceutical production facilities across the UK. Because the skills and facilities required for producing ^68^Ga-labelled radiotracers are similar to those required for conventional ^99m^Tc-labelled radiotracers, RPUs have expanded into ^68^Ga-labelled radiotracers, driven by the availability of generators and radiopharmaceutical kits with MA and commissioning of [^68^Ga]Ga-SSR. Commercial suppliers provide a large volume of commissioned clinical PET radiotracers; additionally, several NHS- and university-run PET radiochemistry facilities provide both commissioned and research radiotracers for clinical studies. Continued investment in new and upgraded facilities has expanded opportunities for radiotracer production, but the site closure reported also indicates insecurity. Few radiotracers are currently available at more than one site, suggesting a need for collaboration, technology transfer and simpler production methods, to enable larger pan-UK multicentre studies. Moreover, if multicentre CT(IMP)s are required, more sites must obtain MIA(IMP) licences.

### Commissioning landscape in the UK

In the UK, clinical commissioning within nuclear medicine requires recommendations in NICE guidelines or NICE technology appraisal guidance or commissioning by NHS Specialised Services. For all routes, the requirement is to show clinical effectiveness of interventions through improvements in patient outcomes. MRT is well suited to this because changes in patient outcomes are core aims of MRT clinical trials. In contrast, diagnostic radiotracers do not directly alter the health of a patient. Consequently, clinical studies assessing them have focused on disease detection and change in clinical management, producing little evidence about whether these management changes affect patient outcome. It appears that evidence for improvement in patient outcomes, with longer-term outcome data, will henceforth be required for clinical adoption of diagnostic radiotracers by the routes described. Increased costs associated with recruiting much larger numbers of patients and monitoring them over years, and other additional hurdles, may explain why such studies are currently rare for diagnostic radiotracers. It is unfortunate that the assessment of diagnostic radiotracers is more stringent than that of medical devices (i.e. NICE diagnostics assessment programme, which accounts for specific considerations and complications of assessing the clinical effectiveness of a diagnostic test).

### Concluding remarks

The route to clinical commissioning in the UK, whether for novel radiopharmaceuticals or new indications for existing radiotracers, depends on (1) whether it is a diagnostic, companion diagnostic or MRT; (2) whether the clinical indications are for rare or orphan diseases; and (3) whether MA has been granted. We have identified three key considerations to support clinical commissioning:

Partnering with industry: Commercialisation of radiopharmaceuticals is preferable if they are to be commissioned. Early partnership with industry provides an opportunity to scope commercial viability and a source of funding for CT(IMP)s and subsequent MA and NICE technology appraisal. There has been strong commercial interest in MRT recently. Particularly noteworthy is the NOVARTIS acquisition of AAA and Endocyte for their MRT technologies, Lutathera and [^177^Lu]Lu-PSMA-617, respectively. Industry partnership may be more difficult for diagnostic agents, but opportunities can arise for companion diagnostics for therapeutic interventions.

Evidence for clinical effectiveness: Clinical effectiveness is a key requirement for commissioning. For diagnostics, trials intended to support MA are unlikely to have gathered suitable evidence for clinical effectiveness, and further trials will be required. Solely showing change in patient management is unlikely to adequately prove clinical effectiveness. Ideally, changes in outcome measured in QALYs should be shown, allowing costs per QALY gained to be compared to current standard of care. Such trials need not be conducted in the UK, but their results must apply to NHS practice.

Evidence for cost-effectiveness: Economic analyses are conducted by NICE for technology assessments and during the impact assessment stage of specialised services commissioning. For NICE to recommend treatment as cost-effective, the cost per QALY gained should be <£30 000. Prioritisation for commissioning within Specialised Services is competitive; interventions with the highest clinical effectiveness for the lowest cost will be prioritised for funding.

## Acknowledgements

We gratefully acknowledge all those who completed the NCITA survey and expert advice received from Dr. Mark Lambert.

We gratefully acknowledge financial support from the Cancer Research UK National Cancer Imaging Translational Accelerator Award (C4278/A27066). MTM acknowledges funding from Cancer Research UK Career Establishment Award (C63178/A24959). EOA acknowledges funding from UK Medical Research Council (MR/N020782/1) Imperial College Experimental Cancer Medicines Centre and NIHR Biomedical Research Centre. SFB acknowledges support from the National Institute for Health Research and Social Care (NIHR) [RP-2016-07-001] and King’s College London and UCL Comprehensive Cancer Imaging Centre funded by CRUK and EPSRC in association with the MRC and Department of Health and Social Care (England). This work was also supported by funding from the EPSRC Programme Grant [EP/S032789/1]; Wellcome/EPSRC Centre for Medical Engineering [WT 203148/Z/16/Z] and National Institute for Health Research (NIHR) Biomedical Research Centre at Guy’s and St Thomas’ NHS Foundation Trust and King’s College London. The views expressed are those of the author(s) and not necessarily those of the NHS, the NIHR or the Department of Health. For the purpose of Open Access, the author has applied a CC BY public copyright licence to any Author Accepted Manuscript version arising from this submission.

J.D.Y., M.J.O., M.T.M., P.J.B. and E.O.A. conceived and designed the report. J.D.Y., M.J.O., M.S.C., M.T.M. and P.J.B. designed the survey and J.D.Y. analysed results. J.D.Y., M.J.O. and M.T.M. conceived and designed the database search and J.D.Y. implemented and analysed it. W.L.W., S.F.B. and G.C. helped conceive and design the commissioning landscape review and provided expert advice. J.D.Y., M.J.O., W.L.W., M.S.C., G.C., S.F.B., M.T.M., P.J.B. and E.O.A. prepared, edited and approved the final manuscript.

### Conflicts of interest

There are no conflicts of interest.

## Supplementary Material



## References

[1] NHS England and NHS Improvement. Diagnostic Imaging Dataset Annual Statistical Release 2019/20 Version 1.0 (Internet). 2020. https://www.england.nhs.uk/statistics/wp-content/uploads/sites/2/2020/10/Annual-Statistical-Release-2019-20-PDF-1.4MB.pdf. [Accessed 9 December 2020]

[2] Scottish Clinical Imaging Network. PET-CT Waiting and Reporting Times (Internet). 2020. https://www.scin.scot.nhs.uk/scin-pet-ct-waiting-reporting-times/. [Accessed 9 December 2020]

[3] Welsh Health Specialised Services Committee. Specialised Services Commissioning Policy: Positron Emission Tomography Version 6.0 (Internet). 2020. https://whssc.nhs.wales/commissioning/whssc-policies/cancer/cp50a-positron-emission-tomography-pet-pdf/. [Accessed 9 December 2020]

[4] Rojas B McGowan DR Guy MJ Tipping J Aldridge M Gear J. Eighty percent more patients in 10 years of UK molecular radiotherapy: Internal Dosimetry Users Group survey results from 2007 to 2017. Nucl Med Commun. 2019; 40:657–661. 31058745 10.1097/MNM.0000000000001020 PMC6587230

[5] Hart D Wall BF. UK Nuclear Medicine Survey 2003-2004. Nucl Med Commun. 2005; 26:937–946. 16208170 10.1097/01.mnm.0000184939.28994.f9

[6] Drzezga A Souvatzoglou M Eiber M Beer AJ Fürst S Martinez-Möller A . First clinical experience with integrated whole-body PET/MR: comparison to PET/CT in patients with oncologic diagnoses. J Nucl Med. 2012; 53:845–855. 22534830 10.2967/jnumed.111.098608

[7] Cherry SR Jones T Karp JS Qi J Moses WW Badawi RD. Total-body PET: maximizing sensitivity to create new opportunities for clinical research and patient care. J Nucl Med. 2018; 59:3–12. 28935835 10.2967/jnumed.116.184028 PMC5750522

[8] Inter-Collegiate Standing Committee on Nuclear Medicine. Evidence-based indications for the use of PET-CT in the United Kingdom 2016 (Internet). 2016. https://www.rcr.ac.uk/publication/evidence-based-indications-use-pet-ct-united-kingdom-2016. [Accessed 26 October 2020]

[9] NHS England. Report for the oxfordhsire health overview and scrutiny: provision of PET-CT services (Internet). 2019. https://mycouncil.oxfordshire.gov.uk/documents/s45274/JHO_APR0419R06 - Provision of PET-CT Services.pdf. [Accessed 15 July 2020]

[10] Buscombe J. The Future of Molecular Radiotherapy Services in the UK. Clin Oncol (R Coll Radiol). 2021; 33:137–143. 33272748 10.1016/j.clon.2020.11.012

[11] Intercollegiate Standing Committee on Nuclear Medicine. Molecular radiotherapy: guidance for clinicians. Second Edition (Internet). 2014. https://www.rcr.ac.uk/system/files/publication/field_publication_files/BFCO(14)2_Molecular_Radiotherapy_Guidance.pdf. [Accessed 26 October 2020]

[12] Medicines and Healthcare Products Regulatory Agency. Is it a clinical trial of a medicinal product? (Internet). 2015. https://www.gov.uk/government/uploads/system/uploads/attachment_data/file/317952/Algothrim.pdf. [Accessed 2021 January 8].

[13] Medicines Act 1968 (Chapter 67) (Internet). 2015. https://www.legislation.gov.uk/. [Accessed 13 January 2021]

[14] National Institute for Health and Care Excellence. Developing NICE guidelines: the manual. 2014. http://www.nice.org.uk/article/pmg20. 26677490

[15] The licensing of medicines in the UK. Drug Ther Bull. 2009; 47:45–48. 19357299 10.1136/dtb.2009.03.0012

[16] European Commision Health and Food Safety Directorate-General. Volume 2A Procedures for marketing authorisation Chapter 1 Marketing Authorisation Revision 11 (Internet). 2019. https://ec.europa.eu/health/sites/health/files/files/eudralex/vol-2/vol2a_chap1_en.pdf. [Accessed 16 December 2020]

[17] Royal Pharmaceutical Society and the NHS Pharmaceutical Quality Assurance Committee. In: Beaney AM, editor. Quality Assurance of Aseptic Preparation Services: Standards. Part A, Fifth Edition. (Internet). London, UK: Royal Pharmaceutical Society; 2016. pp. 110–113. www.rpharms.com/qaaps%0Ahttp://www.rpharms.com/support-pdfs/rps---qaaps-standards-document.pdf.

[18] BNMS Radiopharmaceutical Sciences Group and the UK Radiopharmacy Group. Guidelines for the provision of radiopharmacy services in the UK. 2017. https://www.bnms.org.uk/page/UKRGGuidelines. [Accessed 5 December 2020]

[19] Invicro A Konica Minolta Company. invicro.com (Internet). https://invicro.com/capabilities/radiochemistry/. [Accessed 2021 March 19].

[20] The Medicines and Healthcare products Regulatory Agency. Statutory guidance Current MHRA fees (Internet). 2021. https://www.gov.uk/government/publications/mhra-fees/current-mhra-fees. [Accessed 2021 May 05].

[21] Directive 2001/83/EC of the European Parliament and of the Council of 6 November 2001 on the Community code relating to medicinal products for human use (Internet). 2001. https://eur-lex.europa.eu.

[22] National Institute for Health and Care Excellence. NICE Guidance - Lutetium (177Lu) oxodotreotide for treating unresectable or metastatic neuroendocrine tumours - Technology appraisal guidance (Internet). 2018. https://www.nice.org.uk/guidance/ta539. [Accessed 29 October 2020] 41411462

[23] National Institute for Health and Care Excellence. Guide to the methods of technology appraisal 2013 (Internet). 2013. 10.1183/13993003.01815-2018. [Accessed 29 October 2020] 27905712

[24] National Institute for Health and Clinical Excellence. Diagnostics Assessment Programme Manual (Internet). Online. 2011. http://www.nice.org.uk/media/A0B/97/DAPManualFINAL.pdf. [Accessed 16 December 2020] 27466648

[25] National Institute for Health and Clinical Excellence; Center for Health Technology Evaluation; Medical Technologies Evaluation Programme. NICE Medtech Innovation Briefings -Interim Process and Methods Statement (Internet). 2014. https://www.nice.org.uk/Media/Default/About/what-we-do/NICE-advice/Medtech-innovation-briefings/MIB-interim-process-methods-statement.pdf. [Accessed 16 December 2020]

[26] National Institute for Health and Care Excellence. Nice Advice - Axumin for functional imaging of prostate cancer recurrence - Medtech innovation briefing. 2019. https://www.nice.org.uk/advice/mib172. [Accessed 29 October 2020]

[27] Scottish Clinical Imaging Network. Scottish Clinical Imaging Network (Internet). 2017. https://www.scin.scot.nhs.uk/. [Accessed 2020 December 9].

[28] Scottish Clinical Imaging Network. National Services Division PET-CT Review of Indications Report. 2017. pp. 1–24. https://www.scin.scot.nhs.uk/wp-content/uploads/2017/08/PET-CT-Review-of-Indications-2016-Report-V2-1.pdf. [Accessed 9 December 2020]

[29] Scottish Clinical Imaging Network. Scottish guidelines on the use of ^18^F-FDG PET/CT scanning. 2020. pp. 1–5. https://www.scin.scot.nhs.uk/wp-content/uploads/2020/11/NSD610-005.01.pdf. [Accessed 9 December 2020]

[30] Scottish Clinical Imaging Network. Indications for use of ^18^F-FDG PET/CT in the management of patients with colorectal cancer. 2020. pp. 1–2. https://www.scin.scot.nhs.uk/wp-content/uploads/2020/11/NSD610-005.02.pdf. [Accessed 9 December 2020]

[31] Scottish Clinical Imaging Network. Indications for the use of ^68^Gallium psma pet ct in. 2020. pp. 5–7. https://www.scin.scot.nhs.uk/wp-content/uploads/2020/11/NSD610-005.03.pdf. [Accessed 9 December 2020]

[32] Scottish Clinical Imaging Network. Indications for the use of ^18^F-FDG PET/CT in oesophageal and oesophagogastric junction (OGJ) cancer. 2020. pp. 4–5. https://www.scin.scot.nhs.uk/wp-content/uploads/2020/11/NSD610-005.04.pdf. [Accessed 9 December 2020]

[33] Scottish Clinical Imaging Network. Indications for the use oof ^18^F-FDG PET CT in lung cancer. 2020. pp. 3–5. https://www.scin.scot.nhs.uk/wp-content/uploads/2020/11/NSD610-005.05.pdf. [Accessed 9 December 2020]

[34] Scottish Clinical Imaging Network. Indications for the use of ^18^F-FDG PET CT imaging in the management of head and neck cancer. 2020. https://www.scin.scot.nhs.uk/wp-content/uploads/2020/11/NSD610-005.06.pdf. [Accessed 9 December 2020]

[35] Health and Social Care Act 2012 (Chapter 7) (Internet). 2015. https://www.legislation.gov.uk/. [Accessed 6 January 2021]

[36] NHS England. Manual for Prescibed Specialised Services 2018/19 (Internet). 2018. https://www.england.nhs.uk/wp-content/uploads/2017/10/prescribed-specialised-services-manual.pdf.

[37] NHS England. NHS Commissioning Board Clinical Commissioning Policy Statement: Positron Emission Tomography Computed Tomography (PET-CT) Guidelines (all ages) (Internet). (2015). https://www.england.nhs.uk/wp-content/uploads/2018/08/Positron-emission-tomography-Computed-tomography-guidelines-all-ages.pdf.

[38] Intercollegiate Standing Committee on Nuclear Medicine. Evidence-based indications for the use of PET-CT in the UK 2013. 2013. http://www.nemcb.cz/upload/files/EBM_PET_indications.pdf. [Accessed 26 October 2020]

[39] NHS England. NHS England Specialised Commissioning Service Development Policy (Internet). 2017. https://www.england.nhs.uk/publication/specialised-commissioning-service-development-policy-and-process/.

[40] NHS England. Clinical commissioning work programmes (Internet). 2021. https://www.england.nhs.uk/publication/clinical-commissioning-policy-work-programme/. [Accessed 2021 April 29].

