# Supplementary material for: An overview of nuclear medicine research in the UK and the landscape for clinical adoption
Source: Nucl Med Commun. 2021 Jul 19;42(12):1301–12. doi: 10.1097/MNM.0000000000001461 (PMC8584216; doi:10.1097/MNM.0000000000001461)
Supplement: Supplementary file 1 [file nmc-42-1301-s001.pdf]

An Overview of Nuclear Medicine Research in the UK and the Landscape for Clinical Adoption

Supplementary information

Jennifer D. Young

jennifer.d.young@kcl.ac.uk

School of Biomedical Engineering and Imaging Sciences, King's College London, London, United Kingdom

National Cancer Imaging Translational Accelerator, Cancer Research UK

Maite Jauregui-Osoro

m.jauregui-osoro@imperial.ac.uk

baKingdom

National Cancer Imaging Translational Accelerator, Cancer Research UK

Wai-Lup Wong

wailup.wong@nhs.net

Mount Vernon Cancer Centre, Mount Vernon Hospital, Northwood, United Kingdom

Margaret S. Cooper

margaret.cooper@kcl.ac.uk

School of Biomedical Engineering and Imaging Sciences, King's College London, London, United Kingdom

Gary Cook

gary.cook@kcl.ac.uk

School of Biomedical Engineering and Imaging Sciences, King's College London, London, United Kingdom

King's College London and Guy's and St Thomas' PET Centre, King's College London, King's Health Partners, London, United Kingdom

National Cancer Imaging Translational Accelerator, Cancer Research UK

Sally F. Barrington

sally.barrington@kcl.ac.uk

School of Biomedical Engineering and Imaging Sciences, King's College London, London, United Kingdom

King's College London and Guy's and St Thomas' PET Centre, King's College London, King's Health Partners, London, United Kingdom

Michelle T. Ma

michelle.ma@kcl.ac.uk

School of Biomedical Engineering and Imaging Sciences, King's College London, London, United Kingdom

National Cancer Imaging Translational Accelerator, Cancer Research UK

Philip J. Blower

philip.blower@kcl.ac.uk

School of Biomedical Engineering and Imaging Sciences, King's College London, London, United Kingdom

National Cancer Imaging Translational Accelerator, Cancer Research UK

Eric O. Aboagye*

*Corresponding Author

eric.aboagye@imperial.ac.uk

ORCiD https://orcid.org/0000-0003-2276-6771

Department of Surgery & Cancer, Division of Cancer, Imperial College London, London, United Kingdom

National Cancer Imaging Translational Accelerator, Cancer Research UK

Table 1: Nuclear medicine imaging procedures. This table shows the number of nuclear medicine imaging procedures (i.e. gamma-scintigraphy, SPECT and PET-CT scans) performed within NHS England each year for the last 5 years[1]. In addition, the number of PET-CT scans performed by NHS Scotland[2] and NHS Wales (GIG Cymru)[3] in the 2019-2020 financial year are also listed.

	**NHS England**	**NHS England**	**NHS England**	**NHS Scotland**	**NHS Wales**
**Financial Year**	Gamma-scintigraphy	SPECT Scans	PET-CT Scans	PET-CT Scans	PET-CT Scans
**2015-2016**	432755	25900	97990		
**2016-2017**	423860	35420	132760		
**2017-2018**	417460	40015	154270		
**2018-2019**	421650	45365	177330		
**2019-2020**	396350	45110	199585	7448	3758
**% PET-CT Scans**			94.7%	3.5%	1.8%

Table 2: The 390 unique nuclear medicine studies identified from the database search. NIHR = National Institute for Health Research Clinical Research Network portfolio, NCT = ClinicalTrials.gov database and ISTRCN = ISRCTN registry.

Database	Database Reference	Study Name
NIHR	15698	REPLICA - Reversible hypoxia in lung cancer (version 1.0)
NIHR	16176	Late onset depression: Correlates of dopaminergic function
NIHR	16285	Assessment of 18F-Sodium Fluoride PET/CT as a measure of calcification
NIHR	17110	Brain glutamate-GABA system in ASD
NIHR	17360	FAB-O study
NIHR	17361	The endocannabinoid system and brain function
NIHR	17381	Imaging in Transforming Gliomas
NIHR	17391	REASURE (Radium-223: Evaluation of Activity and SUrrogate REsponse)
NIHR	17642	18F-FMC and MRS in Gliomas
NIHR	17692	18F-FAABULOUS
NIHR	17904	MRC National Survey of Health and Development Neuroimaging substudy
NIHR	18094	Neo-AEGIS
NIHR	18326	iTIMM
NIHR	18356	STRATA
NIHR	18492	GENFI IMPETus Tau PET imaging pilot study
NIHR	18754	The role of phosphodiesterase 10A in early de novo sporadic PD
NIHR	18820	IN-CVD
NIHR	18870	Imaging biomarkers in lung cancer
NIHR	18884	Amyloid PET imaging in the timely diagnosis of Alzheimer's disease
NIHR	18921	In vivo Tau pathology in PSP and CBD
NIHR	18942	FDHT
NIHR	19008	Chronic neuroinflammation following blast TBI
NIHR	19094	BF4
NIHR	19185	NMDA PET in TBI
NIHR	19364	TARGET Trial
NIHR	19427	Surgery and Alzheimer's pathology
NIHR	19520	Study examining the effect of Etanercept on inflammation in the brain
NIHR	19551	The PRE18FFIR Study
NIHR	19580	NEST: NMDA-receptors in Epilepsy, Stroke and TBI
NIHR	19650	ICH-IMAGE
NIHR	19719	EORTC 1217
NIHR	19735	Chemotherapy induced caspase activation in lymphoma
NIHR	19796	Prednisolone and coronary artery disease
NIHR	19937	SEL-I-METRY
NIHR	19972	[11C]CFN PET and fMRI in interictal depression
NIHR	19997	A study of brain amyloid and tau deposition using Florbetapir and T807
NIHR	20039	A PET study in the early phase of psychosis
NIHR	20065	The Brain Imaging In Dementia study (BRAIID)
NIHR	20184	SARON
NIHR	20275	The role of buspirone in LIDs in patients with Parkinson's disease
NIHR	20319	Tiagabine challenge [11C]Ro15 4513 PET in Alcohol Dependence
NIHR	20320	Myelin changes in RRMS patients
NIHR	20371	Astroglial activation, ß-amyloid deposition and neuronal activity
NIHR	20586	Is alcohol dependence associated with increased 18F Amyvid binding?
NIHR	20630	GLP-1 receptor expression in CHI
NIHR	20654	CARBON
NIHR	20704	Imaging Hypoxia in Cerebral Tumours (version 1)
NIHR	20841	EAGLE FM
NIHR	30339	SUPErB
NIHR	30446	Investigating the role of glutamate in brain function
NIHR	30450	In vivo investigations of familial Parkinson's disease
NIHR	30720	PRE18FFIR-IMAGE
NIHR	30749	PADMMA
NIHR	30819	The role of I2BS in Huntington's disease
NIHR	30825	Plerixafor kinetics and radiolabelled neutrophils
NIHR	31043	Tau PET in traumatic brain injury
NIHR	31280	Comparison of quantitative fMRI with FDG-PET in epilepsy
NIHR	31303	PET CT analysis of restenosis after lower limb revascularisation
NIHR	31382	I2PETPG – Imidazoline2 Binding Site in patient group
NIHR	31388	PRE18FFIR-REPRO

NIHR	31442	Predicting outcome from adrenalectomy.
NIHR	31561	Dopamine release during epileptic seizures
NIHR	31584	PRE18FFIR-TIME
NIHR	31690	Biomarkers and Outcomes in CAA (BOCAA)
NIHR	31711	The HIV brain amyloid study
NIHR	31867	CXCR2 inhibition and coronary heart disease / CICADA
NIHR	31962	The REMAP study
NIHR	32003	Neurodegeneration in Aging Down Syndrome (NiAD)
NIHR	32044	Multimodal Imaging in Lewy Body Disorders v1
NIHR	32076	MyxofibroSarcoma staging using PET-MRI: comparison to standard MRI
NIHR	32079	LIFTD-PIN
NIHR	32147	The role of SV2A in PD and PD Dementia
NIHR	32177	Epilepsy: TSPO imaging
NIHR	32325	Synaptic and neural function
NIHR	32353	BBB permeability and Neuro-Inflammation in SVD
NIHR	32503	The role of I2BS in Parkinson’s disease
NIHR	32539	Measuring cerebral protein synthesis rates in Alzheimer's disease
NIHR	32545	REVAMP:Response evaluation in myeloma using 18F-FDG PET/MRI
NIHR	32905	F-MISO PET in borderline resectable pancreatic cancer
NIHR	32970	Tau Evaluation in Neurodegeneration and Dementia Research (TENDeR)
NIHR	32985	Imaging carotid plaque inflammation using GE180-PET/MRI
NIHR	33003	OPERA Study V3.1 - Radiation dose escalation in rectal adenocarcinoma
NIHR	33166	Validation of PET-MRI for cardiovascular disease
NIHR	33175	SCLC trial with nivolumab and ipilimumab in limited disease (STIMULI)
NIHR	33325	MIND-MAPS-PD
NIHR	33381	TSPO PET Imaging in GBM
NIHR	33755	BIVISTA
NIHR	33913	MRI in Lung Cancer Patients
NIHR	33937	SST receptor quantitation - NODAGA-JR11 PET/CT in chest NET
NIHR	34394	HERPET
NIHR	34583	Obesity-related cardiovascular risk and bariatric surgery
NIHR	34687	TSPO PET as a measure of post-stroke brain inflammation
NIHR	34767	PETReA
NIHR	35090	Neurobiology of cognitive function
NIHR	35147	Deep and Frequent Phenotyping
NIHR	35223	STRESSGAP (FEP) version 1
NIHR	35444	ANIMATE
NIHR	35448	Correlation of pre- and post-operative cancer imaging techniques
NIHR	35524	PHAGO-PET
NIHR	35601	STILE version 1.0
NIHR	35741	Boston HF SCS PET Scan
NIHR	35809	Imaging leukocyte accumulation in cancers
NIHR	35889	Effect of food on fatty acid oxidation
NIHR	36010	PET-MR perfusion study
NIHR	36018	PET-MRI Imaging in Patients with Acute Neurovascular Syndrome
NIHR	36230	The contribution of brown adipose tissue to energy expenditure
NIHR	36240	PHERGain
NIHR	36284	Badimon chamber assessment of Al18F-ENC2015 human thrombus binding
NIHR	36294	Characterising the Lung cancer Inflammatory response
NIHR	36296	Choline uptake by brown adipose tissue
NIHR	36300	ARGO
NIHR	36328	Novel Cardiac PET-MR Acquisition and Reconstruction Techniques
NIHR	36347	CCHIRAL version 1
NIHR	36352	PET-MRI Imaging in Patients with Symptomatic Carotid Artery Stenosis
NIHR	36384	PROSPER Study
NIHR	36719	NTAD
NIHR	37078	Simplification of Low Level Internal Dosimetry (SOLLID)
NIHR	37153	Molecular Imaging in Infective Endocarditis with PET-hybrid imaging
NIHR	37293	Amyloid imaging in PREVENT (AIP)
NIHR	37329	MATCH
NIHR	37382	MIND-MAPS AD
NIHR	37493	Imaging brain pathology in dementia using PET and MRI Version 2.0
NIHR	37524	SV2a evaluation in neurodegenerative research
NIHR	37616	Dementias Platform UK MR-PET network harmonisation study
NIHR	37957	PET-MR imaging of coronary atherothrombosis
NIHR	38532	[11C]-(R)-PK11195 PET in metastatic prostate cancer

NIHR	38844	Mood and brain serotonin function
NIHR	38923	STELLAR
NIHR	38976	Neuroreceptor regulation of brain structure and function
NIHR	39017	AMYPAD WP3 - Diagnostic and Patient Management Study
NIHR	39236	18F MicroPet and OPG RANKL in aortic stenosis
NIHR	39382	NEPTUNE
NIHR	39522	People's experiences of low iodine diets
NIHR	39609	A new type of “double scan” for epilepsy in tuberous sclerosis
NIHR	39690	18F Sodium Fluoride PET CT in patients with Acute Aortic Syndrome
NIHR	39725	PRiMAL
NIHR	39763	TeTra
NIHR	39878	Evaluation of the Impact of Developments in PET Methodology
NIHR	40137	INIRA 2 - Biomarkers of neoangiogenesis in Rheumatoid Arthritis
NIHR	40854	IP2 - ATLANTA
NIHR	40952	Functional Assessment of Bone Metastases 2 (FABB2)
NIHR	41135	The RUPTURE study, version 1.0
NIHR	41270	CA125 PET/MRI
NIHR	41290	APOLLO
NIHR	41304	iMarkHD: In Vivo Longitudinal Imaging of HD Pathology
NIHR	41837	In Vivo Imaging of COPD with technetium labelled antibodies (INVICTA)
NIHR	41840	The AoRTAS Study
NIHR	41885	18F-FDG PET/MRI to assess vascular inflammation following MI
NIHR	42016	BioThrombus
NIHR	42036	MIND-MAPS-ALS
NIHR	42080	Glucose-enhanced MRI for detection of altered glucose transport
NIHR	42165	DIRECT
NIHR	42229	INOVATE
NIHR	42266	Ketosis in FDG metabolism in known malignancy
NIHR	42375	Dementias Platform UK Cohort Tau Imaging (DPUK Tau) 1.0
NIHR	42490	FaR-RMS
NIHR	42805	COCONUT (Version 1.0)
NIHR	42913	INSPIRE
NIHR	43462	AMYPAD Prognostic and Natural History Study (PNHS)
NIHR	43629	Identification of biomarkers associated with renal obstruction
NIHR	43762	ORCHESTRA trial
NIHR	43959	ORACLE
NIHR	45262	PETNECK 2
NIHR	45280	Measuring Fatty acid oxidation in cerebral metastases using 18F-FPIA
NIHR	45297	Imperial APC - Version 1.0
NIHR	45338	Siderophores for imaging infection using 68Ga-DFO v1.0
NIHR	45482	Deep MR-only RT
NIHR	46216	PET/MR scanning to quantify brown adipose tissue activity
NIHR	46736	mRCC therapy response by FPIA PET/CT
ISRCTN	ISRCTN10246848	VARIANT: A multicentre randomised feasibility trial of implementing a biomarker-guided personalised treatment in patients with advanced prostate cancer
ISRCTN	ISRCTN10760016	A study of the drugs AZD2014 and rituximab in relapsed or refractory diffuse large B-cell lymphoma
ISRCTN	ISRCTN11499979	Does the planning tool â€œMulti-Criteria Optimisation (MCO)â€� reduce the radiation dose received to the chest wall when planning a lung cancer radiotherapy treatment, in comparison to traditional planning methods?
ISRCTN	ISRCTN11659025	PORTEC-4a: Randomised trial of standard or molecular profile-based recommendation for radiotherapy after surgery for women with early stage endometrial cancer
ISRCTN	ISRCTN12676704	Biological magnetic resonance imaging parameters in cancer
ISRCTN	ISRCTN13626902	The ACCEPT study
ISRCTN	ISRCTN13656673	Depletion of serum Amyloid P Component in Alzheimer's Disease
ISRCTN	ISRCTN13680280	Standard open radical cystectomy (ORC) versus robotically assisted radical cystectomy (RARC)
ISRCTN	ISRCTN13797354	Imaging cerebral neuro-inflammation in acute and chronic cerebrovascular disease: a predictor of outcome and biomarker for guiding treatment (IN-CVD)
ISRCTN	ISRCTN14545692	Investigating the effect of continuous positive airway pressure on the neuropathology of obstructive sleep apnoea
ISRCTN	ISRCTN14616801	A trial investigating whether suppressing the immune system with azathioprine slows the progression of Parkinsonâ€™s disease
ISRCTN	ISRCTN15483452	Switching off leakage and inflammation in small brain blood vessels
ISRCTN	ISRCTN16424014	A phase III trial of intensity-modulated proton beam therapy versus intensity-modulated radiotherapy for multi-toxicity reduction in oropharyngeal cancer
ISRCTN	ISRCTN18092347	Detection of hypoxia (low oxygen) in lung cancer using imaging

ISRCTN	ISRCTN31697115	Kidney function assessment with finger-prick blood tests in different people and different settings
ISRCTN	ISRCTN32667607	A study using a response-based combination therapy of rituximab and ibrutinib in patients with post-transplant lymphoproliferative disorder (PTLD)
ISRCTN	ISRCTN34690731	PET CT analysis of restenosis after lower limb revascularisation
ISRCTN	ISRCTN42886452	Recovery and survival of stem cell originated red cells
ISRCTN	ISRCTN45536692	Zoledronic acid in the management of malignant pleural mesothelioma
ISRCTN	ISRCTN47127434	A trial to look for markers in the tumour cells and blood which signal that trial treatments are working in a patient with triple negative breast cancer, for whom upfront chemotherapy has not provided the maximum expected benefit: PHOENIX
ISRCTN	ISRCTN52651778	A single site, open label, phase I study to assess the safety and feasibility of foetal cell transplants in the striatum of people with Huntingtonâ€™s disease
ISRCTN	ISRCTN55643149	Scleroderma heart study
ISRCTN	ISRCTN56584901	Evaluation of Prostate-Specific Membrane Antigen (68Ga) PET/CT as a tool to guide treatment choice in patients with high risk prostate cancer
NCT	NCT01795521	LungTech: Stereotactic Body Radiotherapy (SBRT) of Inoperable Centrally Located NSCLC
NCT	NCT02071940	PLX3397 KIT in Acral aNd mucOsal Melanoma
NCT	NCT02110303	DIAMOND - Dual Antiplatelet Therapy to Reduce Myocardial Injury
NCT	NCT02131649	PET/MRI for Men Being Considered for Radiotherapy for Suspected Prostate Cancer Recurrence Post-Prostatectomy
NCT	NCT02145416	ART: Anal Squamous Cell Carcinoma: Investigation of Functional Imaging During chemoRadioTherapy
NCT	NCT02166788	Evaluation of Groin Lymphadenectomy Extent For Metastatic Melanoma
NCT	NCT02179125	Identifying REsponders and Exploring Mechanisms of ACTION of the Endobronchial Coil Treatment for Emphysema
NCT	NCT02179970	To Assess the Safety of Continuous IV Administration of Plerixafor in Patients With Advanced Pancreatic, Ovarian and Colorectal Cancers
NCT	NCT02194842	Phase III Radium 223 mCRPC-PEACE III
NCT	NCT02258451	Study of Radium-223 Dichloride Versus Placebo and Treatment With Exemestane / Everolimus in Subjects With Bone Predominant HER2 (Human Epidermal Growth Factor Receptor 2) Negative Hormone Receptor Positive Metastatic Breast Cancer
NCT	NCT02267889	Ganglionated Plexus Ablation For Treatment of Atrial Fibrillation
NCT	NCT02273271	Evaluation of FLT-PET and DWI-MRI in Patients With NSCLC Treated With a Platinum-based Doublet as Preoperative Chemo
NCT	NCT02278211	Prediction of Recurrent Events With 18F-Fluoride (PREFFIR)
NCT	NCT02310672	REPAIR: Right vEntricular Remodeling in Pulmonary ArterIal hypeRtension
NCT	NCT02316106	A Study to Evaluate 3 Dose Schedules of Daratumumab in Participants With Smoldering Multiple Myeloma
NCT	NCT02323217	I2PETHV - Imidazoline2 Binding Site in Healthy Volunteers
NCT	NCT02361385	PBR28 PET and Inflammatory Arthritis
NCT	NCT02365441	A Randomised Trial of Imatinib Alternating With Regorafenib Compared to Imatinib Alone for the First Line Treatment of Advanced Gastrointestinal Stromal Tumour (GIST)
NCT	NCT02388295	AZD3241 azd, Phase 2, Randomized,12 Week Safety and Tolerability Trial With PET in MSA Patients
NCT	NCT02391116	Phase II Copanlisib in Relapsed/Refractory Diffuse Large B-cell Lymphoma (DLBCL)
NCT	NCT02400229	Diagnostic Imaging Strategies for Patients With Stable Chest Pain and Intermediate Risk of Coronary Artery Disease
NCT	NCT02403102	Imageguided Theranostics in Multiple Myeloma
NCT	NCT02431988	Evaluation of CAR19 T-cells as an Optimal Bridge to Allogeneic Transplantation
NCT	NCT02439086	Prediction of Response to Neoadjuvant Therapy in Rectal Cancer
NCT	NCT02442063	Phase Ib Study of Radium Ra 223 Dichloride in Combination With Paclitaxel in Cancer Subjects With Bone Lesions
NCT	NCT02485691	Cabazitaxel Versus the Switch to Alternative AR-targeted Agent (Enzalutamide or Abiraterone) in Metastatic Castration-resistant Prostate Cancer (mCRPC) Patients Previously Treated With Docetaxel and Who Rapidly Failed a Prior AR-targeted Agent
NCT	NCT02486718	Study to Assess Safety and Efficacy of Atezolizumab (MPDL3280A) Compared to Best Supportive Care Following Chemotherapy in Patients With Lung Cancer [IMpower010]
NCT	NCT02511665	Metformin And Longevity
NCT	NCT02534207	Basmisanil Positron Emission Tomography Study in Japanese Volunteers
NCT	NCT02551614	Neutrophil Imaging in Healthy Subjects Following Lipopolysaccharide or Saline Challenge and in Subjects With Chronic Obstructive Pulmonary Disease
NCT	NCT02551653	Positron Emission Tomography (PET) Study to Evaluate Biodistribution of [11C]-GSK2256098 in Healthy Subjects and Idiopathic Pulmonary Arterial Hypertension (PAH) Patients
NCT	NCT02578940	Fluciclovine (18F) PET/CT in biochemicAL reCurrence Of Prostate caNcer
NCT	NCT02581878	Safety and Tolerability of BAY1862864 Injection in Subjects With Relapsed or Refractory CD22-positive Non-Hodgkin's Lymphoma
NCT	NCT02592707	Study to Evaluate the Safety and Preliminary Efficacy of 177Lu-OPS201 in NETs

NCT	NCT02599480	Assessment of Efficacy of Mirabegron, a New beta3-adrenergic Receptor in the Prevention of Heart Failure
NCT	NCT02600897	A Study of Obinutuzumab, Polatuzumab Vedotin, and Lenalidomide in Relapsed or Refractory Follicular Lymphoma (FL) and Rituximab in Combination With Polatuzumab Vedotin and Lenalidomide in Relapsed or Refractory Diffuse Large B-Cell Lymphoma (DLBCL)
NCT	NCT02612051	First Time in Human (FTIH) Study of GSK3008348 in Healthy Volunteers and Idiopathic Pulmonary Fibrosis Patients
NCT	NCT02628080	Atovaquone as Tumour HypOxia Modifier
NCT	NCT02650076	A Study to Investigate the Receptor Occupancy of SK-1405 in Healthy, Caucasian, Male Subjects
NCT	NCT02658513	Evaluation of Lancet Blood Sampling for Radioiodine Dosimetry in Thyroid Cancer
NCT	NCT02658968	Study of Betalutin for Treatment of Relapsed or Refractory Non-Hodgkin Lymphoma (LYMRIT-37-05)
NCT	NCT02666079	The LightPath® Breast Cancer Study
NCT	NCT02677896	A Study of Enzalutamide Plus Androgen Deprivation Therapy (ADT) Versus Placebo Plus ADT in Patients With Metastatic Hormone Sensitive Prostate Cancer (mHSPC)
NCT	NCT02684708	Second International Inter-Group Study for Classical Hodgkin Lymphoma in Children and Adolescents
NCT	NCT02702388	A Phase 2 Trial of Lenvatinib (E7080) in Subjects With Iodine-131 Refractory Differentiated Thyroid Cancer to Evaluate Whether an Oral Starting Dose of 18 mg Daily Will Provide Comparable Efficacy to a 24 mg Starting Dose, But Have a Better Safety Profile
NCT	NCT02703272	A Safety and Efficacy Study of Ibrutinib in Pediatric and Young Adult Participants With Relapsed or Refractory Mature B-cell Non-Hodgkin Lymphoma
NCT	NCT02703844	Vestibular Stimulation in Parkinson's Disease
NCT	NCT02716987	Study to Determine D-Amino Acid Oxidase Brain Enzyme Occupancy of TAK-831 After Single-Dose Oral Administration
NCT	NCT02730338	INTense ExeRcise for surviVAL Among Men With Metastatic Prostate Cancer (INTERVAL - GAP4)
NCT	NCT02739425	The Efficacy of Sentimag in Detection of Sentinel Node Biopsy
NCT	NCT02741856	Study of Chemoradiotherapy in Oesophageal Cancer Including PET Response and Dose Escalation
NCT	NCT02752178	Peripheral Immunomarker Validation in Treatment-resistant Depression
NCT	NCT02753569	Imaging NSCLC Treatment Response to Immunotherapy
NCT	NCT02807181	SIRT Followed by CIS-GEM Chemotherapy Versus CIS-GEM Chemotherapy Alone as 1st Line Treatment of Patients With Unresectable Intrahepatic Cholangiocarcinoma
NCT	NCT02884206	Efficacy and Safety of LCZ696 Compared to Valsartan on Cognitive Function in Patients With Chronic Heart Failure and Preserved Ejection Fraction
NCT	NCT02884453	Proof-of-concept Study of Ibrutinib in c-MYC and HER2 Amplified Oesophagogastric Carcinoma
NCT	NCT02897739	Pathogenesis of Acute Stress Induced (Tako-tsubo) Cardiomyopathy: Energy Shut-Down or Intense Inflammation?
NCT	NCT02899377	A PH I Pilot Imaging Study to Evaluate Molecular Imaging Methods in HVs and pSS Pts
NCT	NCT02910063	Study to Evaluate Safety and Efficacy of Blinatumomab in Subjects With Relapsed/Refractory (R/R) Aggressive B-Cell NHL
NCT	NCT02935816	Localising Occult Prostate Cancer Metastases With Advanced Imaging TEchniques
NCT	NCT02945904	IS Metomidate PET-CT Superior to Adrenal Venous Sampling in Predicting Outcome From Adrenalectomy in Patients With Primary Hyperaldosteronism
NCT	NCT02951156	Avelumab In Combination Regimens That Include An Immune Agonist, Epigenetic Modulator, CD20 Antagonist and/or Conventional Chemotherapy in Patients With Relapsed or Refractory Diffuse Large B-cell Lymphoma (R/R DLBCL)
NCT	NCT02952625	PET/MR in Radiotherapy for Head and Neck Cancer Pilot
NCT	NCT02956486	A 24-Month Study to Evaluate the Efficacy and Safety of Elenbecestat (E2609) in Subjects With Early Alzheimer's Disease
NCT	NCT02959892	A Single-Dose Positron Emission Tomography (PET) Study to Determine the Effect of TAK-041 on Amphetamine-Induced Dopamine Release in the Central Nervous System (CNS)
NCT	NCT02967536	Investigating the Neuropathology of Obstructive Sleep Apnoea
NCT	NCT02975518	Tracking Endothelial Cells in Arterial Injury
NCT	NCT02976883	[18F]HX4 PET/CT Imaging for Detection of Hypoxia
NCT	NCT02987543	Study of Olaparib (Lynparzaâ„¢) Versus Enzalutamide or Abiraterone Acetate in Men With Metastatic Castration-Resistant Prostate Cancer (PROfound Study)
NCT	NCT02988531	Validation of PET-MRI for Cardiovascular Disease
NCT	NCT02989480	PET-detected Myocardial Inflammation is a Characteristic of Cardiac Sarcoid But Not of ARVC
NCT	NCT02997371	IL-1ra Dose-range Study for Moderate-to-severe TBI Patients
NCT	NCT03003078	A Pilot Study of OncoSilâ„¢ Given to Patients With Pancreatic Cancer Treated With FOLFIRINOX or Gemcitabine+Abraxane
NCT	NCT03006172	To Evaluate the Safety, Tolerability, and Pharmacokinetics of GDC-0077 Single Agent in Participants With Solid Tumors and in Combination With Endocrine and Targeted Therapies in Participants With Breast Cancer
NCT	NCT03011580	Vitamin D to Resolve Inflammation After Tuberculosis (ResolveD-TB)
NCT	NCT03019536	A Study of LY3303560 in Participants With Mild Cognitive Impairment or Alzheimer's Disease

NCT	NCT03022357	Nigrosomal Iron Imaging in Parkinson's Disease
NCT	NCT03023878	Safety and Efficacy of Blinatumomab in Subjects With Newly Diagnosed High-risk Diffuse Large B-Cell Lymphoma
NCT	NCT03029026	The Role of Occult Cardiac Amyloid in the Elderly With Aortic Stenosis.
NCT	NCT03036280	A 24-Month Study to Evaluate the Efficacy and Safety of Elenbecestat (E2609) in Subjects With Early Alzheimer's Disease_
NCT	NCT03036943	Fluciclovine (18F) Imaging of Breast Cancer
NCT	NCT03048760	Magnetic Resonance Imaging (MRI) for the Delineation of Organs At Risk (OAR) and Target Volumes in Lung Cancer Patients
NCT	NCT03049189	Efficacy and Safety of 177Lu-edotreotide PRRT in GEP-NET Patients
NCT	NCT03065816	Scintigraphy Study to Compare the Antireflux Activity of the Z0063 Versus Gaviscon Double Action Tablets, in Healthy Adult Subjects
NCT	NCT03093064	Inflammatory Response In Schizophrenia
NCT	NCT03107988	NANT 2015-02: A Phase 1 Study of Lorlatinib (PF-06463922)
NCT	NCT03131453	A Study of CNP520 Versus Placebo in Participants at Risk for the Onset of Clinical Symptoms of Alzheimer's Disease
NCT	NCT03148795	A Study of Talazoparib in Men With DNA Repair Defects and Metastatic Castration-Resistant Prostate Cancer
NCT	NCT03150056	Dose Escalation and Dose Expansion Study of GSK525762 in Combination With Androgen Deprivation Therapy and Other Agents in Subjects With Castrate-resistant Prostate Cancer
NCT	NCT03161353	Chemotherapy-free Trastuzumab and Pertuzumab in HER2-positive Breast Cancer: FDG-PET Response-adapted Strategy.
NCT	NCT03177187	Combination Study of AZD5069 and Enzalutamide.
NCT	NCT03188666	A Study to Examine the Safety, Tolerability and Effects on Abnormal Bone Formation of REGN2477 in Patients With Fibrodysplasia Ossificans Progressiva
NCT	NCT03198442	Breast PET Feasibility
NCT	NCT03212157	GlucoCEST MRI in Oncology
NCT	NCT03215550	PET-MRI Imaging in Patients With Symptomatic Carotid Artery Stenosis
NCT	NCT03215563	PET-MRI Imaging in Patients With Acute Neurovascular Syndrome
NCT	NCT03245268	International BPA Registry
NCT	NCT03274492	A Study Comparing the Efficacy and Safety of Polatuzumab Vedotin With Rituximab-Cyclophosphamide, Doxorubicin, and Prednisone (R-CHP) Versus Rituximab-Cyclophosphamide, Doxorubicin, Vincristine, and Prednisone (R-CHOP) in Participants With Diffuse Large B-Cell Lymphoma
NCT	NCT03275402	131I-omburtamab Radioimmunotherapy for Neuroblastoma Central Nervous System/Leptomeningeal Metastases
NCT	NCT03284957	Phase 1 / 2 Study of SAR439859 Single Agent and in Combination With Palbociclib in Postmenopausal Women With Estrogen Receptor Positive Advanced Breast Cancer (AMEERA-1)
NCT	NCT03289143	A Study to Evaluate the Efficacy and Safety of Semorinemab in Patients With Prodromal to Mild Alzheimer's Disease
NCT	NCT03294434	Predicting Sites of Tumour Progression in the Invasive Margin of Glioblastomas (PRaM-GBM Study)
NCT	NCT03312712	Validation of the Analysis Methodology Behind the Use of Quantitative 18F-FDG PET/CT to Assess Lung Inflammation
NCT	NCT03318523	Evaluating the Safety, Pharmacokinetics, and Pharmacodynamics of BIIB054 in Participants With Parkinson's Disease
NCT	NCT03327662	Utilising CTC Counts to Optimize Systemic Therapy of Metastatic Prostate Cancer
NCT	NCT03337919	ANIMATE: Phase II Study of Nivolumab Monotherapy for Relapsed/Refractory Hodgkin Lymphoma
NCT	NCT03352089	Positron Emission Tomography / Magnetic Resonance Imaging in Aortic Stenosis
NCT	NCT03355027	Investigating the Lowest Threshold of Vascular Benefits From LDL Cholesterol Lowering in Patients With Stable CV Disease
NCT	NCT03363373	Naxitamab for High-Risk Neuroblastoma Patients With Primary Refractory Disease or Incomplete Response to Salvage Treatment in Bone and/or Bone Marrow
NCT	NCT03399071	Study Title: Peri-operative Immuno-Chemotherapy in Operable Oesophageal and Gastric Cancer
NCT	NCT03405012	Regional Bone Turnover Using 18F-fluoride-PET/CT in HIV-1-infected Men: PETRAM Study
NCT	NCT03405025	Radiofrequency Endoscopic Ablation With Ultrasound Guidance: a Non-surgical Treatment for Aldosterone-producing Adenomas
NCT	NCT03409549	Multi-parametric MRI/Fluorine-18 Fluciclovine PET-CT in Glioblastoma
NCT	NCT03422523	Atezolizumab, Rituximab, Gemcitabine and Oxaliplatin in Patients With Relapsed or Refractory DLBCL Not Suitable for High-dose Therapy
NCT	NCT03437941	Study to Evaluate CORT125281 in Combination With Enzalutamide in Patients With mCRPC
NCT	NCT03443973	Safety and Efficacy Study of Gantenerumab in Participants With Early Alzheimer's Disease (AD)
NCT	NCT03446001	Safety and Efficacy of TRx0237 in Subjects With Alzheimer's Disease Followed by Open-Label Treatment

NCT	NCT03451448	PET MRI in Coronary Artery Disease
NCT	NCT03452228	Safety and Efficacy Following Repeat-Dose of Evinacumab (Anti-ANGPTL3) in Patients With Severe Hypertriglyceridemia (sHTG) at Risk for Acute Pancreatitis
NCT	NCT03458585	Radiation Exposure Awareness From Patients Undergoing Nuclear Medicine Diagnostic Scans
NCT	NCT03473847	Repeatability, Reproducibility and Comparison of Cirrus OCT, RTVue OCT, MS-39 OCT, and Insight 100 VHFDU
NCT	NCT03506997	Trial of Pembrolizumab in Metastatic Castration Resistant Prostate Cancer
NCT	NCT03507452	First-in-human Study of BAY2287411 Injection, a Thorium-227 Labeled Antibody-chelator Conjugate, in Patients With Tumors Known to Express Mesothelin
NCT	NCT03507569	Open Label, Adaptive, Parallel Group PET Study Using RO7017773 And [11C] RO15-4513
NCT	NCT03509428	The Wessex Fit-4-Cancer Surgery Trial
NCT	NCT03510338	Pharmacoscintigraphic Study to Evaluate Two Sildenafil Products
NCT	NCT03511664	Study of 177Lu-PSMA-617 In Metastatic Castrate-Resistant Prostate Cancer
NCT	NCT03526809	Molecular Imaging and Spectroscopy With Stable Isotopes in Oncology and Neurology
NCT	NCT03533283	An Open-Label Phase IB Study of Glofitamab and Atezolizumab or Polatuzumab Vedotin in Adult Patients With Relapsed/Refractory B-Cell Non-Hodgkin's Lymphoma
NCT	NCT03568656	Study to Evaluate CCS1477 in Advanced Tumours
NCT	NCT03580161	Simplification of Low Level Internal Dosimetry
NCT	NCT03593759	Cardiac Sarcoidosis Randomized Trial
NCT	NCT03617588	68Ga-THP-PSMA PET/CT Imaging in High Risk Primary Prostate Cancer or Biochemical Recurrence of Prostate Cancer
NCT	NCT03617666	Avelumab in the Frontline Treatment of Advanced Classical Hodgkin Lymphoma - a Window Study
NCT	NCT03618303	PET-MR Imaging of Coronary Atherothrombosis
NCT	NCT03626571	PET/MR Imaging In Patients With Infective Endocarditis
NCT	NCT03626584	PET/MR Imaging In Patients With Cardiac Amyloidosis
NCT	NCT03644303	Targeted Radiotherapy in Androgen-suppressed Prostate Cancer Patients.
NCT	NCT03647566	18F Sodium Fluoride PET/CT in Acute Aortic Syndrome
NCT	NCT03662750	TSPO PET as a Measure of Post-stroke Brain Inflammation: a Natural History Cohort
NCT	NCT03691064	Post-Authorization Long-Term Safety Study of LUTATHERA
NCT	NCT03705884	PET/MR Imaging In Patients With Cardiac Sarcoidosis
NCT	NCT03716557	A High Frequency Spinal Cord Stimulation PET-CT Scan Study
NCT	NCT03724747	Study to Evaluate the Safety, Tolerability,Pharmacokinetics, and Anti-tumor Activity of a Thorium-227 Labeled Antibody-chelator Conjugate, in Patients With Metastatic Castration Resistant Prostate Cancer
NCT	NCT03731026	The LightPath® and 68Ga-RM2 in Breast Cancer Study
NCT	NCT03732820	Study on Olaparib Plus Abiraterone as First-line Therapy in Men With Metastatic Castration-resistant Prostate Cancer
NCT	NCT03748641	A Study of Niraparib in Combination With Abiraterone Acetate and Prednisone Versus Abiraterone Acetate and Prednisone for Treatment of Participants With Metastatic Prostate Cancer
NCT	NCT03767244	A Study of Apalutamide in Participants With High-Risk, Localized or Locally Advanced Prostate Cancer Who Are Candidates for Radical Prostatectomy
NCT	NCT03782753	Molecular Pathology and Neuronal Networks in LRRK2 Parkinson's Disease
NCT	NCT03790709	ANAVEX2-73 for Treatment of Early Alzheimer's Disease
NCT	NCT03815838	Molecular Imaging of Pituitary Adenomas
NCT	NCT03859895	Zoledronate In the Prevention of Paget's Disease: Long Term Extension
NCT	NCT03887455	A Study to Confirm Safety and Efficacy of BAN2401 in Participants With Early Alzheimer's Disease
NCT	NCT03906045	A Scintigraphy Study of PT010 in COPD Patients
NCT	NCT03914248	Monitoring Large Vessel Vasculitis With PET/MR Imaging
NCT	NCT03920371	Evaluation of a Prototype Hand Held Hybrid Gamma Camera
NCT	NCT03927209	A Study in Healthy Men to Test the Effects of Different Doses of BI 1467335 on MAO-B Activity in the Brain.
NCT	NCT03934372	Safety and Efficacy of Ponatinib for Treatment of Pediatric Recurrent or Refractory Leukemias or Solid Tumors
NCT	NCT03935672	PEARL PET-based Adaptive Radiotherapy Clinical Trial
NCT	NCT03940092	Sodium (23Na) MRI for Tumour Characterisation and Assessment of Therapy Response in Breast Cancer
NCT	NCT03943966	In-vivo Thrombus Imaging With 18F-GP1, a Novel Platelet PET Radiotracer
NCT	NCT03996473	Study to Test the Safety and How Radium-223 Dichloride an Alpha Particle-emitting Radioactive Agent Works in Combination With Pembrolizumab an Immune Checkpoint Inhibitor in Patients With Stage IV Non-small Cell Lung Cancer With Bone Metastases
NCT	NCT04038957	A Clinical Study to Investigate the Effect of an Investigational Drug as an Added Medication to an Antipsychotic, in Adults With Schizophrenia, as Measured Positron Emission Tomography (PET) Imaging
NCT	NCT04063826	PET-MR Study of Fatty Liver

NCT	NCT04071691	PET Imaging of Giant Cell and Takayasu Arteritis
NCT	NCT04073797	PET Imaging of Inflammation and Lipid Lowering Study
NCT	NCT04073810	Residual Inflammation and Plaque Progression Long-term Evaluation
NCT	NCT04073875	18F-GP1 PET-CT to Detect Bioprosthetic Aortic Valve Thrombosis
NCT	NCT04078191	Comparison of Tc 99m Tilmanocept Quantitative Imaging With Immunohistochemical (IHC) Analysis of CD206 Expression in Synovial Tissue of Subjects With Rheumatoid Arthritis (RA)
NCT	NCT04082286	Yttrium-90 Anti CD66 Monoclonal Antibody in Childhood Relapsed/Refractory Leukaemia
NCT	NCT04083118	Assessment of Risk in Thoracic Aortopathy Using 18F-Sodium Fluoride
NCT	NCT04097535	Measuring Fatty Acid Oxidation in Gliomas Using 18F-FPIA PET/MRI
NCT	NCT04147819	A First in Human Study of BAY2701439 to Look at Safety, How the Body Absorbs, Distributes and Excretes the Drug, and How Well the Drug Works in Participants With Advanced Cancer Expressing the HER2 Protein
NCT	NCT04182204	A Study to Evaluate the Safety and Efficacy of Polatuzumab Vedotin in Combination With Rituximab, Gemcitabine and Oxaliplatin Compared to Rituximab, Gemcitabine and Oxaliplatin Alone in Participants With Relapsed or Refractory Diffuse Large B-Cell Lymphoma
NCT	NCT04226937	DLBCL Interim Response Evaluation for Customised Therapy
NCT	NCT04240223	A Study to Investigate the Use of Delayed Release Tablets for Colonic Delivery of Brilacidin in Healthy Volunteers
NCT	NCT04241601	Low-dose Interleukin-2 for the Reduction of Vascular Inflammation in Acute Coronary Syndromes - IVORY
NCT	NCT04242459	Optimising Radiation Therapy in Head and Neck Cancers Using Functional Image-Guided Radiotherapy and Novel Biomarkers
NCT	NCT04245839	A Study to Evaluate the Efficacy and Safety of JCAR017 in Adult Subjects With Relapsed or Refractory Indolent B-cell Non-Hodgkin Lymphoma (NHL)
NCT	NCT04276272	D4 Choline Breast PET/CT
NCT	NCT04285996	IMaging Pilot Study of the αvβ6 Integrin Radiotracer [18F]-A20FMDV2 in PAtients With Solid Cancer Types (IMPACT)
NCT	NCT04307953	Saracatinib Trial TO Prevent FOP
NCT	NCT04315246	177Lu-DTPA-Omburtamab Radioimmunotherapy for Leptomeningeal Metastasis From Solid Tumors
NCT	NCT04327817	Multifidus PET Scan Study
NCT	NCT04362436	TheraSphere Selective Internal Radiation Therapy (SIRT) as Treatment for Neuroendocrine Tumours With Liver Mets
NCT	NCT04383158	Alveolar Socket Healing With and Without PRGF
NCT	NCT04391244	Investigating National Solutions for Personalised Iodine-131 Radiation Exposure
NCT	NCT04412369	Multi-modality Imaging & Immunophenotyping of COVID-19 Related Myocardial Injury
NCT	NCT04436406	PDL1 Expression in Cancer (PECan Study).
NCT	NCT04462263	Study to Investigate the Receptor Occupancy of HTL0014242 Using [18F] FPEB in Healthy Male Subjects
NCT	NCT04497844	A Study of Niraparib in Combination With Abiraterone Acetate and Prednisone Versus Abiraterone Acetate and Prednisone for the Treatment of Participants With Deleterious Germline or Somatic Homologous Recombination Repair (HRR) Gene-Mutated Metastatic Castration-Sensitive Prostate Cancer (mCSPC)
NCT	NCT04507698	INTERVAL - Intense Exercise Trial for Men With Metastatic Castrate-Resistant Prostate Cancer
NCT	NCT04521361	A Study to Assess How Radium-223 Distributes in the Body of Patients With Prostate Cancer Which Spread to the Bones
NCT	NCT04529018	CETO First in Human Trial
NCT	NCT04538014	A Study in Healthy Men to Investigate Uptake and Distribution of Lu AF88434 in the Brain
NCT	NCT04577716	Predicting Endoleaks Following Endovascular Aortic Aneurysm Repair Using 18F-Sodium Fluoride
NCT	NCT04592341	A Study to Evaluate the Pharmacodynamic (PD) Effects of Once Weekly Administration of Gantenerumab in Participants With Early Alzheimer's Disease (AD)
NCT	NCT04597125	Study to Learn More About a Drug Called Radium-223 Dichloride (Xofigo) Given and Compared With the Standard Dose of a New (Novel) Anti-hormonal Therapy (NAH) in Adult Male Participants With Prostate Gland Cancer Which Spread to the Bone

Table 3: Radiotracers identified from the database search and the nuclear medicine service area they were assigned to for analysis.

Radiotracer			
**Gamma-scintigraphy/SPECT**	**PET**		**MRT**
[^{123}I]DaTscan	[^{11}C]BU99008	[^{18}F]FES	[^{131}I]NaI
[^{123}I]FPCIT	[^{11}C]Carfentanil	[^{18}F]Florbetaben	[^{131}I]Omburtamab
[^{123}I]MIBG	[^{11}C]CFN	[^{18}F]Florbetapir	[^{177}Lu]Lu-oxodotreotide /[^{177}Lu]Lu-DOTA-TATE
[^{99m}Tc]Tc-albumin	[^{11}C]DASB	[^{18}F]Flortaucipir	[^{177}Lu]Lu-DTPA-Omburtamab
[^{99m}Tc]Tc-DPD	[^{11}C]DASB	[^{18}F]FLT	[^{177}Lu]Lu-Edotreotide
[^{99m}Tc]Tc-DTPA	[^{11}C]GSK2256098	[^{18}F]Fluciclovine	[^{177}Lu]Lu-Lilotomab Satetraxetan
[^{99m}Tc]Tc-HMPAO	[^{11}C]L-deprenyl-D2	[^{18}F]Fluoroazomycin arabinoside ([^{18}F]FAZA)	[^{177}Lu]Lu-OPS201
[^{99m}Tc]Tc-MDP	[^{11}C]Leucine	[^{18}F]Fluoro-L-thymidine	[^{177}Lu]Lu-PSMA-617
[^{99m}Tc]Tc-MAG3	[^{11}C]LuAF88434	[^{18}F]Fluoromisonidazole ([^{18}F]FMISO)	[^{223}Ra]RaCl_2
[^{99m}Tc]Tc-maraciclatide	[^{11}C]MePPEP	[^{18}F]Flutemetamol	[^{227}Th]Th-BAY1862864
[^{99m}Tc]Tc-NM01	[^{11}C]Methionine	[^{18}F]FPEB	[^{227}Th]Th-BAY2287411
[^{99m}Tc]Tc-sulfur colloid	[^{11}C]Metomidate	[^{18}F]FPIA	[^{227}Th]Th-BAY2315497
[^{99m}Tc]Tc-sulfur colloid standard meal	[^{11}C]MK-8278	[^{18}F]GE179	[^{227}Th]Th-BAY2701439
[^{99m}Tc]Tc-tilmanocept	[^{11}C]pBR28	[^{18}F]GE180	[^{90}Y]Y-Anti CD66
[^{81m}Kr]Kr gas ventilation	[^{11}C]PHNO	[^{18}F]GE226	[^{90}Y]Y-Resin microspheres
[^{57}Co]Co transmission scan	[^{11}C]PK11195	[^{18}F]GLP1	
VQ scan	[^{11}C]RO15-4513	[^{18}F]GP1	
	[^{11}C]SA4503	[^{18}F]GTP1	
	[^{11}C]UCB-J	[^{18}F]HX4	
	[^{18}F]BCPP-EF	[^{18}F]ICMT11	
	[^{18}F]BF_4	[^{18}F]NaF	
	[^{18}F]CETO	[^{18}F]PGM299	
	[^{18}F]fluoromethyl-[1,2-2H4]-choline ([^{18}F]D4-FCH)	[^{18}F]T807	
	[^{18}F]DPA-714	[^{62}Cu][Cu(ATSM)]	
	[^{18}F]Fallypride	[^{68}Ga]Ga-DFO	
	[^{18}F]FBAA20FMDV2	[^{68}Ga]Ga-DOTA-TATE	
	[^{18}F]Fluorocholine	[^{68}Ga]Ga-PSMA-11	
	[^{18}F]FDG	[^{68}Ga]Ga-RM2	
	[^{18}F]FDHT	[^{68}Ga]Ga-THP-PSMA	
	[^{18}F]FDOPA	[^{89}Zr]Zr-DFO-B43.13	

Figure 1: **A:** Proportion of nuclear medicine studies from each of the three service areas, Gamma-scintigraphy or SPECT imaging, PET imaging or Molecular Radiotherapy. **B:** proportion of nuclear medicine studies within the three main specialities Cancer, Neurology and Cardiovascular (if a study did not fit any of these it was designated as other). **C:** proportion of nuclear medicine studies that received industry funding.

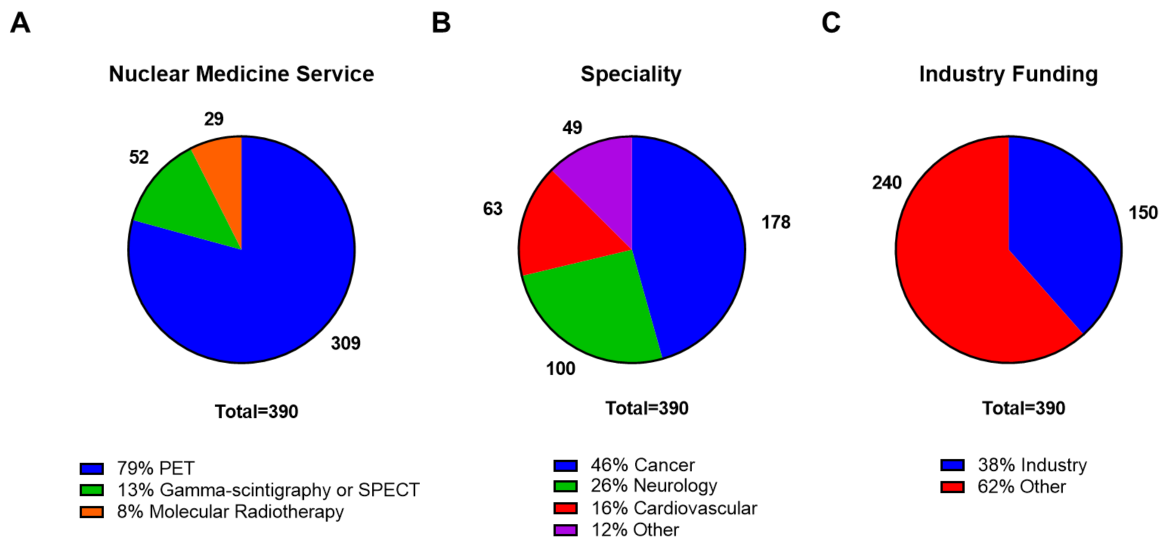

Figure 2: Proportion of observational studies, non-CT(IMP) interventional study, CT(IMP)s where the radiotracer is not the IMP, and CT(IMP)s where the radiotracer is the IMP for **A:** PET studies **B:** Gamma-scintigraphy or SPECT studies and **C:** MRT studies. **D:** proportion of PET studies that utilise each category of PET radiotracers. **E:** proportion of gamma-scintigraphy and SPECT studies that utilise each category of radiotracers. **F:** proportion of MRT studies that utilise each category of MRT radiotracer.

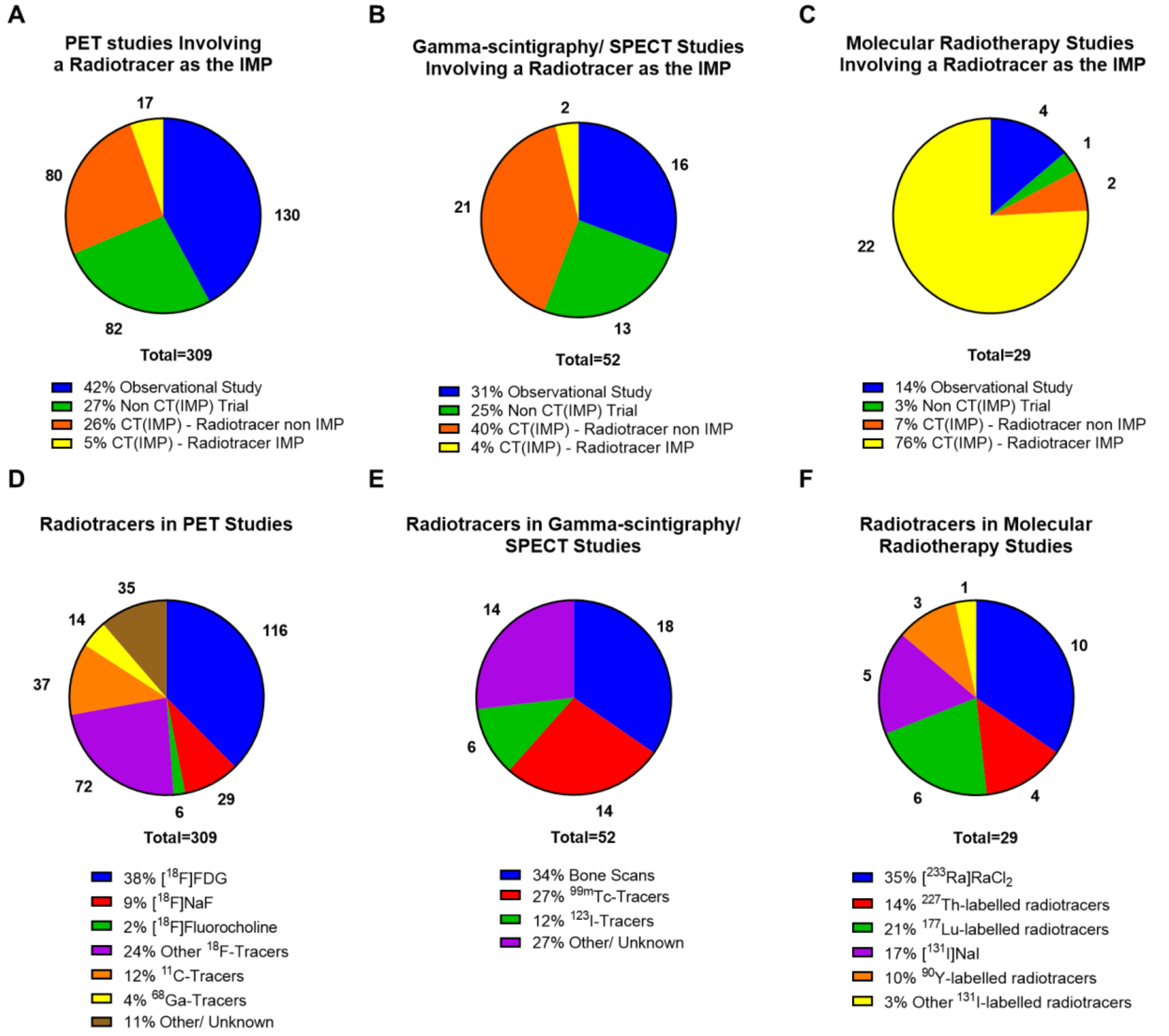

Table 4: Description of Manufacturer Licences in the UK.

Manufacturer Licence - Manufacturing and Importation Authorisation (MIA)	• In the UK, a manufacturer licence (also known as Manufacturing and Importation Authorisation - MIA) granted by the UK licencing authority is required in order to manufacture products for which MA has been granted.<br>• The obligations of the MIA holder, including compliance with the principles and guidelines of GMP, are defined in the Human Medicines Regulations 2012.<br>• Sites are inspected regularly and issued GMP certificates upon passing the inspection.<br>• A manufacturer licence must name the responsible Qualified Person (QP), Production Manager and Quality Controller [4]<br>• Once the MA is granted, products produced at the manufacturing sites listed within are labelled as GMP, with the MA number, and can be marketed by the MAH in the specified countries.<br>• Although a company may hold a UK MA for a radiopharmaceutical product and be marketing it in the UK, they are not obliged to use a UK-based manufacturing site and do not need to be able to deliver the product to the whole country nor meet national demand.
Manufacturer "Specials" Licence (MS)	• Article 5(1) of Directive 2001/83/EC Community Code Relating to Medicinal Products for Human Use [5] allows countries to waive the need for MA to fulfil "special needs".<br>• In the UK, medicines without a MA can be supplied by manufacturers that hold a manufacturer "specials" licence (MS) from the UK licencing authority.<br>• The MHRA inspects the site regularly to ensure that facilities are operating in compliance with GMP regulations and other conditions of the licence.<br>• A MS licence must name the responsible Production Manager and Quality Controller for the site but there is no requirement for a QP.<br>• MHRA inspection of sites holding manufacturer "specials" licence is site-specific, as opposed to product-specific, and the products manufactured cannot be labelled as GMP.<br>• Restrictions that come with products made under a manufacturer "specials" licence include<br>(i) products must be used for clinical "special needs" of an individual patient as determined by the prescriber [6].<br>(ii) products cannot be marketed or advertised [6].<br>(iii) products cannot be used instead of those with a MA for reasons of cost, convenience or operational needs unless the product with a MA becomes unavailable (no longer obtainable from normal distribution channels in a reasonable time).
Manufacturer Licence for Investigational Medicinal Products - Manufacturing and Importation Authorisation for Investigational Medicinal Products (MIA(IMP))	• In the UK, a manufacturer licence for investigational medicinal products (also known as Manufacturing and Importation Authorisation for Investigational Medicinal Products - MIA(IMP)) is required from the UK licencing authority in order to manufacture products for investigation in a clinical trial.<br>• The obligations of the MIA(IMP) holder, including compliance with the principles and guidelines of GMP, are defined in the EU clinical trials directive 2001/20/EC.<br>• Sites must comply with GMP and are inspected regularly by the MHRA.<br>• A manufacturer licence for investigational medicinal products must name the responsible QP, Production Manager and Quality Controller.<br>• It is the QP's responsibility to ensure that each batch has been manufactured and checked in compliance with the product specification file, good manufacturing practice for medicinal products for human use regulations, and the process outlined in the clinical trial application and IMP dossier before it is released for use to the clinical trial sponsor.

Figure 3: Survey sent to UK Radiotracer Production Facilities and Services.

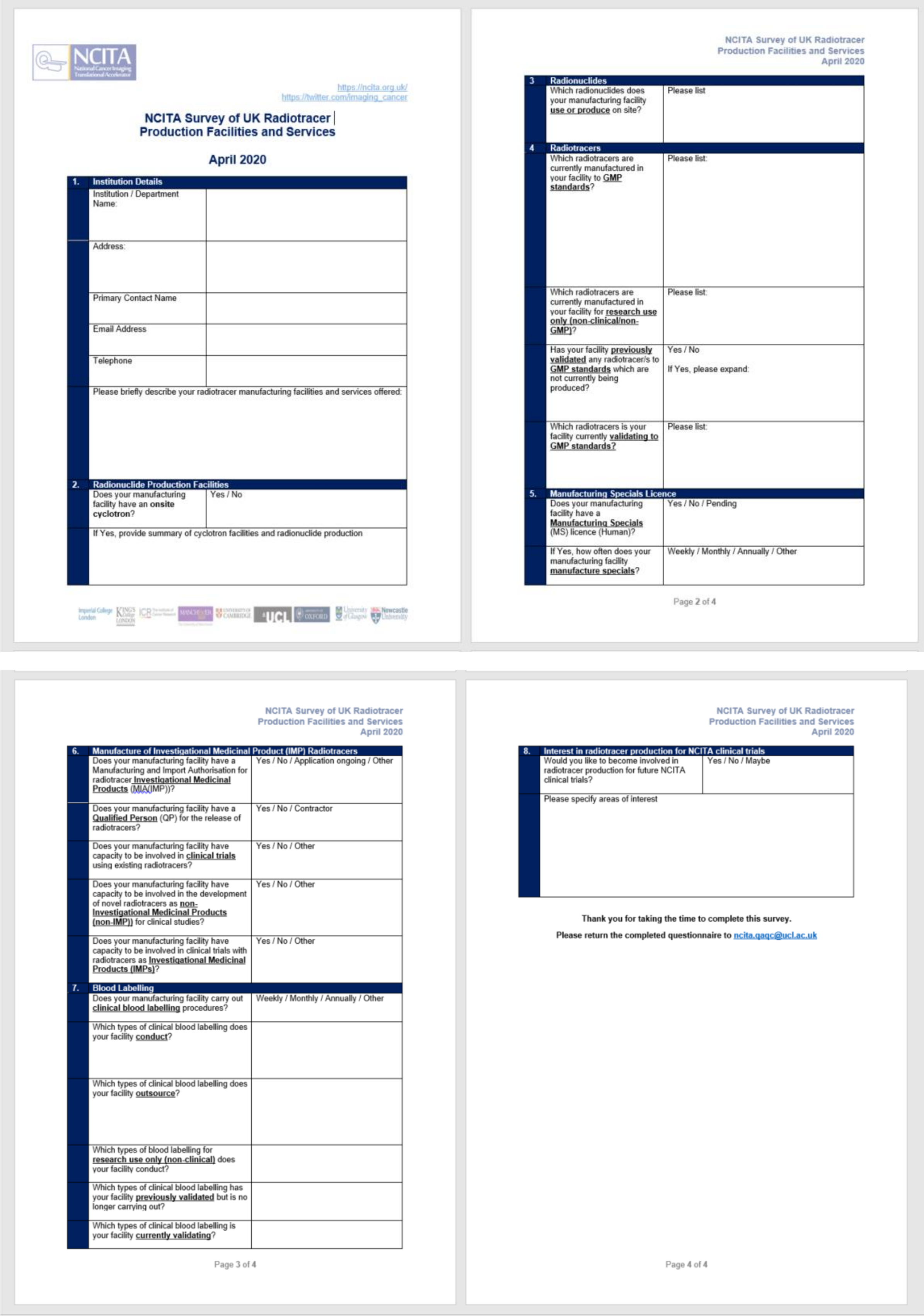

NCITA National Cancer Imaging Translational Accelerator

https://ncita.org.uk/
https://twitter.com/imaging_cancer

NCITA Survey of UK Radiotracer Production Facilities and Services

April 2020

**1.**	**Institution Details**	
	Institution / Department Name:	
	Address:	
	Primary Contact Name	
	Email Address	
	Telephone	
	Please briefly describe your radiotracer manufacturing facilities and services offered:	
**2.**	**Radionuclide Production Facilities**	
	Does your manufacturing facility have an **onsite cyclotron**?	Yes / No
	If Yes, provide summary of cyclotron facilities and radionuclide production	

Imperial College London, King's College London, ICR, Manchester, University of Cambridge, UCL, University of Oxford, University of Glasgow, Newcastle University

NCITA Survey of UK Radiotracer Production Facilities and Services April 2020

**3**	**Radionuclides**	
	Which radionuclides does your manufacturing facility **use or produce** on site?	Please list
**4**	**Radiotracers**	
	Which radiotracers are currently manufactured in your facility to **GMP standards**?	Please list:
	Which radiotracers are currently manufactured in your facility for **research use only (non-clinical/non-GMP)**?	Please list:
	Has your facility **previously validated** any radiotracer/s to **GMP standards** which are not currently being produced?	Yes / No<br>If Yes, please expand:
	Which radiotracers is your facility currently **validating to GMP standards?**	Please list:
**5.**	**Manufacturing Specials Licence**	
	Does your manufacturing facility have a **Manufacturing Specials** (MS) licence (Human)?	Yes / No / Pending
	If Yes, how often does your manufacturing facility **manufacture specials**?	Weekly / Monthly / Annually / Other

Page 2 of 4

NCITA Survey of UK Radiotracer Production Facilities and Services April 2020

**6.**	**Manufacture of Investigational Medicinal Product (IMP) Radiotracers**	
	Does your manufacturing facility have a Manufacturing and Import Authorisation for radiotracer **Investigational Medicinal Products** (MIA(IMP))?	Yes / No / Application ongoing / Other
	Does your manufacturing facility have a **Qualified Person** (QP) for the release of radiotracers?	Yes / No / Contractor
	Does your manufacturing facility have capacity to be involved in **clinical trials** using existing radiotracers?	Yes / No / Other
	Does your manufacturing facility have capacity to be involved in the development of novel radiotracers as **non-Investigational Medicinal Products (non-IMP)** for clinical studies?	Yes / No / Other
	Does your manufacturing facility have capacity to be involved in clinical trials with radiotracers as **Investigational Medicinal Products (IMPs)**?	Yes / No / Other
**7.**	**Blood Labelling**	
	Does your manufacturing facility carry out **clinical blood labelling** procedures?	Weekly / Monthly / Annually / Other
	Which types of clinical blood labelling does your facility **conduct**?	
	Which types of clinical blood labelling does your facility **outsource**?	
	Which types of blood labelling for **research use only (non-clinical)** does your facility conduct?	
	Which types of clinical blood labelling has your facility **previously validated** but is no longer carrying out?	
	Which types of clinical blood labelling is your facility **currently validating**?	

Page 3 of 4

NCITA Survey of UK Radiotracer Production Facilities and Services April 2020

**8.**	**Interest in radiotracer production for NCITA clinical trials**	
	Would you like to become involved in radiotracer production for future NCITA clinical trials?	Yes / No / Maybe
	Please specify areas of interest	

Thank you for taking the time to complete this survey.

Please return the completed questionnaire to ncita.qaqc@ucl.ac.uk

Page 4 of 4

Figure 4: Survey sent to UK Commercial Radiotracer Suppliers.

NCITA National Cancer Imaging Translational Accelerator

https://ncita.org.uk/
https://twitter.com/imaging_cancer

NCITA Survey of UK Commercial Radiotracer Suppliers

June 2020

**1.**	**Company Details**	
	Company / Department Name:	
	Address:	
	Primary Contact Name	
	Email Address	
	Telephone	
	Please briefly describe your radiotracer manufacturing facilities and services offered:	
**2.**	**Radionuclide Production Facilities**	
	Do you have cyclotron facilities?	Yes / No
	If Yes, provide summary of your cyclotron facilities and radionuclide production:	

Imperial College London · King's College London · ICR · Manchester · University of Cambridge · UCL · University of Oxford · University of Glasgow · Newcastle University

**3**	**Radionuclides**	
	Which radionuclides does your company supply?	Please list
**4**	**Radiotracers**	
	Which radiotracers do you supply to under a **GMP licence for routine clinical use**?	Please list:
	Which radiotracers do you supply under a **GMP licence for clinical use as specials?**	Please list:
	Which radiotracers do you supply under a **GMP licence for clinical use as IMPs?**	Please list:
	Which radiotracers do you supply for **research use only (non-clinical/non-GMP)**?	Please list:
	Which radiotracers are you **validating** with the aim to supply under your **GMP licence for clinical use** within the next 2 years?	Please list:
**5.**	**Manufacturing Specials Licence**	
	Does your facility have a **Manufacturing Specials** (MS) licence (Human)?	Yes / No / Pending
	If Yes, how often does your facility **manufacture specials**?	Daily/ Weekly / Monthly / Annually / Other

**6.**	**Manufacture of Investigational Medicinal Product (IMP) Radiotracers**	
	Does your facility have a Manufacturing and Import Authorisation for radiotracer **Investigational Medicinal Products** (MIA(IMP))?	Yes / No / Application ongoing / Other
	If Yes, how often does your facility **manufacture IMPs**?	Daily/ Weekly / Monthly / Annually / Other
	Do you have capacity to develop, validate and supply clinical trials with novel radiotracers as **Investigational Medicinal Products (IMPs)**?	Yes / No / Other
	Do you have the capacity to develop, validate and supply novel radiotracers as **non-Investigational Medicinal Products (non-IMP)** for clinical studies?	Yes / No / Other
**7.**	**Interest in radiotracer production for studies adopted by NCITA**	
	Are you interested in supplying radiotracers for the NCITA network?	Yes / No / Maybe
	Please specify areas of interest	

Thank you for taking the time to complete this survey.

Please return the completed questionnaire to ncita.qaqc@ucl.ac.uk

Table 5: A table summarising the responses from NHS and academic PET radiochemistry facilities that use cyclotron produced radionuclides and whether they are currently operational.

PET Radiochemistry Facilities	Completed survey	Currently operational	Manufacturer Licenses
Wales Research and Diagnostic PET Imaging Centre, Cardiff University;	Yes	Yes	MS
John Mallard Scottish PET Centre, NHS Grampian Aberdeen Royal Infirmary, University of Aberdeen;	Yes	No – new cyclotron being installed	NA
PET Tracer Production Unit (PTPU), Newcastle University;	Yes	No – due to open 2021	NA
Wolfson Brain Imaging Centre, University of Cambridge;	Yes	Yes	MS and MIA(IMP)
Centre for Radiopharmaceutical Chemistry, Division of Medicine, University College London;	Yes	Yes	MS and MIA(IMP)
Wolfson Molecular Imaging Centre, University of Manchester;	Yes	No – closed December 2020	NA
PET Research Centre and Molecular Imaging Research Centre, University of Hull and Hull University Teaching Hospitals NHS Trust	Yes	No – due to open 2021	NA
West of Scotland PET Centre; Glasgow	Yes	Yes	MS and MIA(IMP)
Edinburgh Imaging, University of Edinburgh;	Yes	Yes	MS and MIA(IMP)
Positron Emitting Radiopharmaceutical Laboratory, King’s College London	Yes	Yes	MS and MIA(IMP)
Oxford PET Radiochemistry – PROx	No	No – build completed but no opening date	NA

Table 6: RPUs that completed the survey and whether they hold a manufacturer "specials" licence, manufacturer licence for investigational medicinal products or operate under section 10.

RPUs	Completed survey	Manufacturer Licenses
Radiopharmacy Unit, Barts Health NHS Trust	Yes	MS and MIA(IMP)
Radiopharmacy Department, Queen Elizabeth Hospital, Birmingham	Yes	MS
Radiopharmacy, Nuclear Medicine, Addenbrookes Hospital, Cambridge University Hospitals NHS Foundation Trust	Yes	MS
Radiopharmacy Unit, The Christie NHS Foundation Trust	Yes	MS and MIA(IMP)
Radionuclide Dispensary, NHS Greater Glasgow and Clyde	Yes	MS and MIA(IMP)
Guy's Radiopharmacy, Guy's & St Thomas' Foundation NHS Trust	Yes	MS and MIA(IMP)
Radiopharmacy Unit, Nuclear Medicine Centre, Manchester University NHS Foundation Trust	Yes	MS and MIA(IMP)
Radiopharmacy, Churchill Hospital, Oxford University Hospitals NHS Foundation Trust	Yes	MS
Radiopharmacy, Nuclear Medicine, Royal Free London NHS Foundation Trust	Yes	MS and MIA(IMP)
Radiopharmacy Department, The Royal Marsden NHS Foundation Trust	Yes	MS and MIA(IMP)
Radiopharmacy Department, University Hospitals Southampton NHS Foundation Trust	Yes	MS and MIA(IMP)
Nuclear Medicine Department, Singleton Hospital, Swansea	Yes	MS
Department of Nuclear medicine, The Newcastle upon Tyne Hospitals NHS Foundation Trust	Yes	MS
Leeds Teaching Hospitals NHS trust	No	Section 10
University of Hull and Hull University Teaching Hospitals NHS Trust Radiopharmacy	Yes	MS

Table 7: Sites manufacturing MRT radiotracers under their manufacturer "specials" licence or manufacturer licence for investigational medicinal products.

**RPU**	**MRT Radiotracers**
Radiopharmacy Department, University Hospitals Southampton NHS Foundation Trust	[^{177}Lu]Lu-PSMA<br>[^{227}Th]Th-PSMA (antibody)<br>[^{227}Th]Th-mesothelium<br>[^{90}Y]Y-anti-CD66 (and [^{111}In]In-anti-CD66 for screening)
Radiopharmacy, Nuclear Medicine, Royal Free London NHS Foundation Trust	[^{90}Y]Y-anti-CD66 (and [^{111}In]In-anti-CD66 for screening)
Radiopharmacy Department, The Royal Marsden NHS Foundation Trust	[^{177}Lu]Lu-PSMA<br>[^{90}Y]Y-DOTA-TATE (and [^{111}In]In-DOTA-TATE for screening)

Table 8: Radiotracers no longer available for human use in the UK due to Wolfson Molecular Imaging Centre, University of Manchester closing in 2020.

Radionuclide	Radiotracer
Carbon-11	[^{11}C]Antisense compound<br>[^{11}C]DASB<br>[^{11}C]Diprenorphine<br>[^{11}C]Flumazenil<br>[^{11}C]MDL100907<br>[^{11}C]PK11195<br>[^{11}C]Temozolomide<br>[^{11}C]Verapamil
Iodine-124	[^{124}I]mIBG
Fluorine-18	[^{18}F]FAZA<br>[^{18}F]Florbetapir ([^{18}F] AV-45)<br>[^{18}F]GE-180<br>[^{18}F]UCB-H
Oxygen-15	[^{15}O]Carbon monoxide<br>[^{15}O]Water

Table 9: The ^{99m}Tc-labelled radiotracers that are being made under manufacturer “specials” licence in the UK and the sterile kits granted marketing authorisation that these can be produced from. * Finland MA = 11229, Denmark MA = DK R 1171.

Radiopharmaceutical Produced from Kit	Commercial Kit Name	MA
[^{99m}Tc]Tc-colloid	Hepatate<br>NANOCOLL<br>NanoScan<br>Nanotop	PL 22879/0002<br>PL 16991/0001<br>PL 40129/0002<br>PL 45925/0002
[^{99m}Tc]Tc-DMSA	RENOCIS	PL 11876/0008
[^{99m}Tc]Tc-DPD (butedronate)	Teceos	No UK MA *
[^{99m}Tc]Tc-DTPA	PENTACIS<br>TechneScan DTPA	PL 11876/0011<br>PL 12288/0011
[^{99m}Tc]Tc-hydroxymethylene diphosphonate (HDP) (oxidronate)	Technescan HDP<br>OSTEOCIS	PL 12288/0012<br>PL 11876/0006
[^{99m}Tc]Tc-exametazime (HMPAO)	Ceretec<br>Stabilised Ceretec<br>Medi-Exametazime	PL 00221/0126<br>PL 00221/0136<br>PL 40129/0001
[^{99m}Tc]Tc-HYNIC-Tyr3 -Octreotide	Tektrotyd	PL 45925/0001
[^{99m}Tc]Tc-MAA (microagregated albumin)	PULMOCIS<br>TechneScan LyoMAA	PL 11876/0009<br>PL 12288/0013
[^{99m}Tc]Tc-mertiatide (MAG3)	Technescan MAG3<br>Renoscan MAG3<br>IEL MAG3	PL 12288/0014<br>PL 27151/0001<br>PL 45925/0002
[^{99m}Tc]Tc-mebrofenin (HIDA)	CHOLEDIUM	PL 22879/0001
[^{99m}Tc]Tc-methylene diphosphonate (MDP) (medronate)	DRAXIMAGE	PL 29620/0002
[^{99m}Tc]Tc-sestamibi	CARDIOVIS<br>DRAXMIBI<br>STAMICIS	PL 34397/0001<br>PL 29620/0003<br>PL 11876/0019
[^{99m}Tc]Tc-pyrophosphate	Technescan PYP	PL 12288/0015
[^{99m}Tc]Tc-tetrofosmin	MYOVIEW<br>Tetrofosmin ROTOP	PL 00221/0142<br>PL 45925/0004

Table 10: Radiopharmaceuticals reported in survey for gamma-scintigraphy or SPECT that are received from suppliers ready for administration.

**Product**	**MAH**	**MA**
DaTScan (Ioflupane (^{123}I))	GE Healthcare B.V.	EU/1/00/135/001
Gallium (Ga-67) Citrate Injection	Curium Netherlands B.V.	PL 12288/0018
AdreView (Iobenguane (^{123}I))	GE Healthcare Limited	PL 00221/0140
MIBG (I-123) Injection (Iobenguane (^{123}I))	Mallinckrodt Medical B.V.	PL 12288/0007
Meta-Iodobenzylguanidine (131I) for Diagnostic Use	GE Healthcare Limited	PL 00221/0124
Indium (111In) DTPA Injection	Curium Netherlands B.V.	PL 12288/0005
Thallium [^{201}Tl] chloride injection	CIS bio international	PL 11876/0012
SeHCAT 370 kBq capsules	GE Healthcare Limited	PL 00221/0105
Sodium Iodide (I-123) Injection	Mallinckrodt Medical B.V	PL 12288/0009

Table 11: Table of radiotracers reported in survey for MRT that have MA and are delivered to hospitals ready for clinical administration.

MA	MAH	Product
EMEA/H/C/002653	Bayer AG	Xofigo (Radium [^{223}Ra] dichloride)
EMEA/H/C/004123	Advanced Accelerator Applications	Lutathera (Lutetium [^{177}Lu] oxodotreotide, ^{177}Lu]Lu-DOTA-[Tyr3]-octreotate, [^{177}Lu]Lu-DOTA-TATE)
PL 00221/0102	GE Healthcare Limited	THERACAP131 37 MBq-5.55 GBq hard capsules
PL 00221/0112	GE Healthcare Limited	Sodium Iodide (131I) Diagnostic Capsules 0.333-3.7 MBq capsules, hard
PL 00221/0113	GE Healthcare Limited	Sodium Iodide (131I) Injection 74 MBq/mL and 925 MBq/mL solution for injection
PL 00221/0125	GE Healthcare Limited	Meta-Iodobenzylguanidine (131I) for Therapeutic Use 185 – 740 MBq/mL solution for infusion or solution for injection.
PL 00221/0127	GE Healthcare Limited	METASTRON 37 MBq/mL solution for injection
PL 12288/0010	Mallinckrodt Medical B.V	Sodium Iodide (I131) Capsules T

Table 12: ^{18}F-labelled radiotracers that can be supplied by commercial suppliers, and their marketing authorisation status.

Supplier	Radiotracer	Marketing Authorisation Status	Marketing Authorisation Holder	Manufacturer Site
Alliance Medical Radiopharmacy Ltd.	[^{18}F]FDG (Fludeoxyglucose)<br>[^{18}F]Florbetaben (Neuraceq)	Marketing Authorisation	PL 22443/0001 & PL 22443/0002<br>EMEA/H/C/002553	UK<br>UK
	[^{18}F]Fluoroethylcholine ([^{18}F]FEC)<br>[^{18}F]NaF	Unlicensed “Special”	NA<br>NA	UK<br>UK
Siemens Healthcare Ltd;	[^{18}F]FDG (Metatrace)	Marketing Authorisation	PL 45366/0001	UK
PETNET UK	[^{18}F]Fluoromethylcholine ([^{18}F]FMC)<br>[^{18}F]NaF<br>[^{18}F]F-PSMA-1007	Unlicensed “Special”	NA<br>NA<br>NA	UK<br>UK<br>UK
Advanced Accelerator Applications, a Novartis Company	[^{18}F]FDOPA ([^{18}F]6-fluoro-Ldihydroxyphenylalanine, DOPAVIEW)	Marketing Authorisation	PL 35145/0001	France

Table 13: Germanium-68/ gallium-68 generators and radiopharmaceutical kits to make ^{68}Ga-labelled products granted marketing authorisation.

Supplier/ Importer	Marketing Authorisation Holder	Product	Marketing Authorisation
Curium Pharma UK	IRE-ELiT	GalliAd, 0.74 -1.85 GBq, radionuclide generator	PL 43883/0001
Advanced Accelerator Applications	Eckert & Ziegler Radiopharma GmbH	GalliaPharm, 0.74 – 1.85 GBq, radionuclide generator	PL 46734/0001
Advanced Accelerator Applications	Advanced Accelerator Applications	SomaKit TOC (DOTA-TOC)	EMEA/H/C/004140
Advanced Accelerator Applications	Advanced Accelerator Applications	NETSPOT (DOTA-TATE)	208547<br>Initial U.S. Approval: 2016<br>No UK or EC MA

Table 14: Summary of NICE publication types - guidelines, guidance and advice.

**NICE Guidelines** [7]	NICE guidelines make evidence-based recommendations for England on a range of health and care-related subjects [7]. Most relevant to the nuclear medicine community are the NICE guidelines that set out the care and services that are suitable for most people with a specific condition or need, and those about how medicines should be managed in different settings. A typical guideline will describe the patient pathway for a particular disease or condition, make recommendations about how patients should be assessed and then managed or treated, and describe how this may change over time. Explanations of how recommendations were derived and summaries of the evidence are provided alongside guidelines. NICE guidelines cover health and care in England. In other UK countries, decisions on how these guidelines are applied are made by ministers in the Welsh Government, Scottish Government and Northern Ireland Executive. In England, clinical commissioning groups (CCGs), NHS England and healthcare providers have a responsibility to enable NICE guidelines to be applied, despite this not being a legal obligation. NICE guidelines include the use of medicines granted MA. In some circumstances, off-label use of medicines granted UK MA may be recommended, but medicines (including radiopharmaceuticals) that have not been granted UK MA will not be considered for inclusion in guidelines [7].
**NICE Guidance**	NICE Guidance is produced through a number of evaluation programs that evaluate both clinical-effectiveness and cost-effectiveness of new health technologies. The choice of the most suitable evaluation program depends on the type of technology to be evaluated.
**NICE Guidance**<br>New Pharmaceuticals with UK MA	New pharmaceuticals or new indications for their use are typically evaluated through the technology appraisal programme or if indicated for an ultra-orphan disease, the highly specialised technologies programme. All medicinal products and indications considered for these programs must already have been granted UK MA or be expected to receive this within 20 months so that the NICE guidance can be published concurrently with that approval. Technologies that will remain without MA cannot be evaluated. NICE aims to consider all significant drugs and indications, and most technologies are identified by the National Institute for Health Research (NIHR) Innovation Observatory [8]. Technology appraisal is specifically designed to appraise a health technology for a single indication and is based on a review of clinical and economic evidence [9].<br>As of 2019, there is a charge for NICE to conduct technology appraisals or highly specialised technology evaluation. For 2019-2020 this charge ranges from £88,000 to £251,000, depending on the complexity of the appraisal [10]. Although the focus of technology appraisals is typically on new pharmaceuticals or newly licensed indications, there is flexibility for any health technology to be evaluated through this programme.<br>The National Institute for Health and Care Excellence (Constitution and Functions) and the Health and Social Care Information Centre (Functions) Regulations 2013 [11] and the NHS constitution [12] state that when NICE recommends that the NHS should fund new technology by publishing technology appraisal guidance or highly specialised technology guidance, the NHS must implement this within three months, unless NICE specifies a longer implementation period.
**NICE Guidance**<br>New Medical Technologies with CE Marking	Medical technologies with CE marking, or those on course to receive a CE marking within 12 months, are typically evaluated by the NICE medical technologies evaluation programme or the diagnostics assessment programme. This includes medical devices, active medical devices, active implantable medical devices and an *in vitro* diagnostic medical devices. Currently, no charge is associated with products going through the medical technologies evaluation programme or the diagnostics assessment programme. When medical technologies guidance or diagnostics guidance are published, the NHS does not have an obligation to fund the recommendations made by NICE.
**NICE Guidance**<br>New Companion Diagnostics	Companion diagnostics are diagnostic tests that enable the patients that will respond best to new treatments to be identified. The NICE guide to the methods of technology appraisal states that if the sole purpose of the diagnostic test and it is essential for establishing the suitability of patients, then when a NICE technology appraisal is conducted for the therapy, the companion diagnostic can also be included [13,14]. This means the diagnostic test is incorporated into the assessments of clinical and cost-effectiveness. In addition, the diagnostic accuracy of the test for the particular biomarker of treatment efficacy should be examined.<br>Key considerations which influence if the companion diagnostic is included in the technology assessment are:<br>(i) how the companion diagnostic is described in the MA for the therapy.<br>(ii) how it was used in clinical trials.<br>The NICE guide to the methods of technology appraisal says that if there are multiple diagnostic tests available for a therapy, the NICE diagnostics assessment program [13,14] will be used to determine which should be used in the NHS. However, the NICE diagnostics assessment program has been tailored for medical devices and not medicinal products and so it is unclear if this route would be used for radiotracers.
**NICE Advice**	NICE advice is distinct from NICE guidance, and there are three key types of NICE advice: Evidence Summaries, Medtech Innovation Briefings and Key Therapeutic Topics. These provide objective summaries of the information available, are purely advisory and do not constitute a guidance recommendation. They are designed to support commissioners or staff considering using new technologies [15,16,17].

Table 15: A summary of the pharmaceutical tests, pre-clinical tests and clinical trials in the EMA assessment report for Axumin ($[^{18}F]$Fluciclovine) Procedure No. EMEA/H/C/004197/0000 which received EC MA in 2017 [18].

**Pharmaceutical (physico-chemical, biological or microbiological) tests**					
Information on development, manufacture and control of the active substance, precursor and finished product.					
As required by the guideline on radiopharmaceuticals the non radioactive "chemical precursor" for the synthesis of PET radiopharmaceuticals was shown to comply with the Note for Guidance on Summary of Requirements for Active Substances in Part II of the Dossier (CHMP/QWP/297/97 Rev. 1)					
**Preclinical (toxicological and pharmacological) tests**					
In vivo investigation for PK and PD in mice, rat, doga and monkey					
Toxicity studies in rats, dogs and monkey species					
The influence of the major$[^{18}F]$Fluciclovine -related substance anti-1-amino-3-hydroxycyclobutane- carboxylic-acid (OHACBC) on the pharmacodynamic activity and or the uptake of $[^{18}F]$Fluciclovine was investigated in human cancer cell lines (*in vitro*) and in monkey, dog and rat (*in vivo*)					
**Clinical Trials**					
Study No./ Sponsor	Study Design	Population	Number of Subjects	Study drug dose	Endpoints
**Phase 1**					
GE148-001 GE Healthcare Sorensen, 2013 McParland, 2013	Open label, single dose	Healthy volunteers<br><br>Primary prostate cancer	6<br><br>6	Mean dose 154 MBq<br><br>Mean dose 418 MBq	Safety, biodistribution and dosimetry in HVs<br><br>Safety, uptake and retention in biopsy-proven prostate cancer
NMK36-P1 NMP Asano, 2011	Open label, single dose	Healthy volunteers	6	174-201 MBq	Safety, biodistribution and dosimetry
Emory University Nye, 2007	Open label, single dose	Healthy volunteers	6	Mean dose 366 MBq	Safety, biodistribution and dosimetry
**Phase 2**					
GE148-002 GE Healthcare Study report	Open label, single dose	Primary prostate cancer	21	Mean dose 351 MBq	Safety and proof of concept efficacy data (Efficacy and pharmacokinetics analyses were not performed because GE Healthcare terminated the study early for administrative reasons.)
NMK36-PC-201 NMP Study report	Open label, single dose	Primary prostate cancer	10	94-288 MBq	Safety, dose ranging and proof of concept efficacy data
NMK36-PC-202 NMP Study report	Multicentre, open label, single dose	Primary prostate cancer patients at intermediate and high risk of LN involvement and patients with advanced primary disease and known metastases	68	Low dose: 127 ± 10%<br><br>High dose: 270 ± 10% MBq	Efficacy assessment by diagnostic performance vs CT/bone scan; safety
**Phase 3**					
R01 No study report retrieved from the submission	Retrospective collection of data from patients exposed to $[^{18}F]$Fluciclovine for the diagnosis of cancer	Recurrent prostate cancer Primary prostate cancer Breast cancer	713	Mean dose 309 MBq (range 140-485 MBq)	Efficacy vs histopathology standard of truth Comparison to $[^{11}C]$choline Safety
BED001/ Emory University Study report	Open label single dose, comparative study vs $[^{111}In]$In-ProstaScint	Recurrent prostate cancer	115	162 – 485 MBq	Diagnostic performance vs histology
BED001/ Bologna University Study report	Open label single dose, comparative study vs $[^{11}C]$choline	Recurrent prostate cancer	91	~ 370 MBq	Detection rate vs $[^{11}C]$choline

Reader Study					
BED002 Reader study	Blinded image evaluation of [^{18}F]Fluciclovine PET-CT scans from: 1. The R01 Emory University study (biopsy population)<br>2. Bologna University (comparison to [^{11}C]choline)	Recurrent prostate cancer	105<br><br>88	NA	[^{18}F]Fluciclovine PET-CT diagnostic performance vs histopathological standard of truth<br><br>Comparison of [^{18}F]Fluciclovine PET-CT read and onsite [^{11}C]choline PET CT scan read

Table 16: A summary of the pharmaceutical tests, pre-clinical tests and clinical trials in the EMA assessment report for Lutathera ([^{177}Lu]Lu- oxodotreotide) EU/1/17/1226/001 which received EC MA in 2017 [19].

**Pharmaceutical (physico-chemical, biological or microbiological) tests**					
Information on development, manufacture and control of the active substance, precursor and finished product.					
**Preclinical (toxicological and pharmacological) tests**					
In vivo investigation for PK in mice, and cynomolgus monkeys					
Multiple dose pharmacokinetic/toxicokinetic (TK) data were obtained during the course of repeat-dose toxicity studies in mice, rats, and cynomolgus monkeys					
Safety pharmacology studies were performed using the non-radioactive compound [^{175}Lu]Lu-oxodotreotide. Single and repeat dose toxicity studies were performed in rats and dogs.					
**Clinical Trials**					
Study No./ Sponsor	Study Design	Population	No Subjects	Study drug dose	Endpoints
**Phase 1 and 2**					
ERASMUS (MC) Phase 1/2<br><br>MEC 127.545/1993/84<br><br>Brebander et al. 2017 [20]	Single arm	Patients with somatostatin receptor positive gastro-entero-pancreatic neuroendocrine tumours (GEP NETs)	1214	29.6 GBq (4 x 7.4 GBq at 6 to 13-week intervals)	Safety, treatment efficacy, quality of life (EORTC questionnaire), time to progression, progression free survival, PK/biodistribution and dosimetry data.<br><br>Patients monitored until disease progression or death.
**Phase 3**					
NETTER 1<br><br>NCT 01578239<br><br>Strosberg et al. 2017 [21]	Multicentre, stratified, open label, randomised, comparator-controlled, parallel group.<br><br>Phase 3	Patients with inoperable, progressive, somatostatin receptor positive, well-differentiated neuroendocrine **tumours of the small bowel (mid-gut carcinoid tumours)**	229	Lutathera arm:<br>29.6 GBq (4 x 7.4 GBq at 8-week intervals) plus best supportive care (30 mg) octreotide LAR<br><br>Octreotide LAR arm:<br>High dose (60 mg) octreotide LAR	Progression free survival, objective response rate, overall survival, time to progression, tolerability, quality of life (EORTC questionnaire)<br><br>Patients to be followed up for 5 years from the date of randomisation of the final patient (end expected Jan 2021).

Figure 5: Overview of clinical studies used as evidence in the EMA assessment report for Lutathera ([^{177}Lu]Lu-oxodotreotide) EU/1/17/1226/001 which received EC MA in 2017 [19]. Figure reproduced from the same assessment report [19].

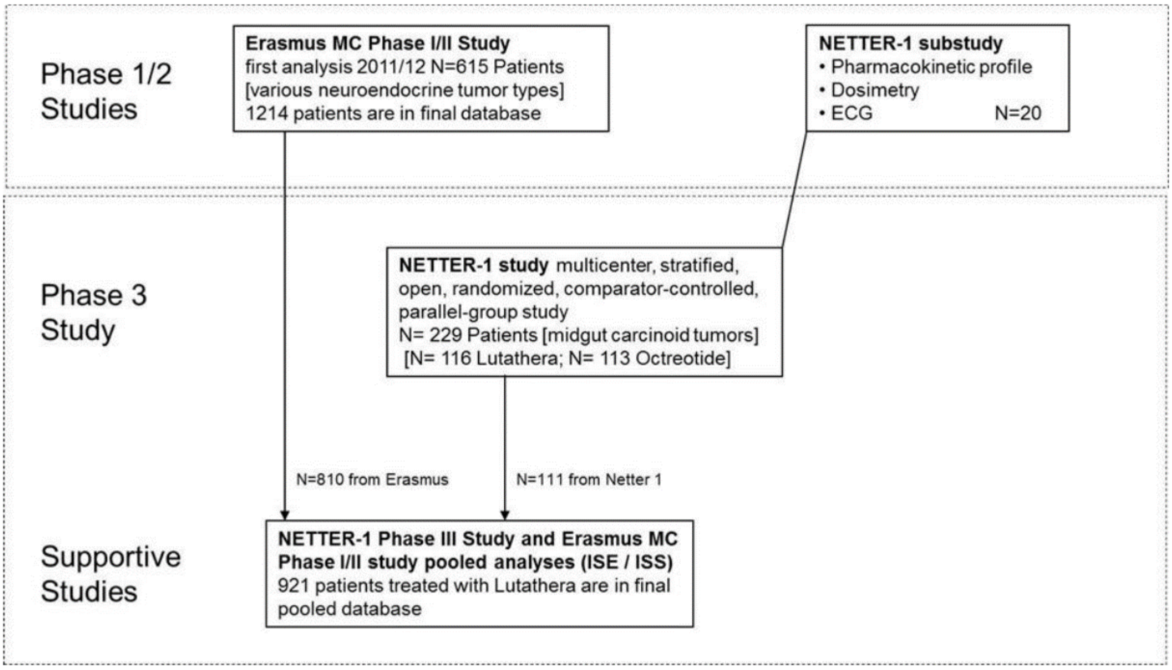

Table 17: Clinical effectiveness data for Lutathera using matched adjusted indirect treatment comparisons as published in the NICE health technology appraisal guidance [22].

**Prolonging overall survival compared to**	**Prolonging overall survival compared to**	**Hazard Ratio**	**95% Confidence interval**	**Lutathera more effective than comparator**
Pancreatic NETs	Best supportive care	0.22	0.10 to 0.50	Statistically significantly
	Everolimus	0.54	0.33 to 0.88	Statistically significantly
	Sunitinib	0.65	0.16 to 2.54	Not statistically significantly
Gastrointestinal NETs	Best supportive care	0.34	0.16 to 0.69	Statistically significantly
	Everolimus	0.55	0.27 to 1.11	Not statistically significantly

Table 18: Evidence used for NICE Medtech innovation briefing on Axumin ([^{18}F]Fluciclovine) 2019 [23].

Study	Name of Paper	Study size, design and location	Key outcomes
Andriole et al. (2019) [24]<br><br>NCT02680041<br><br>(17 study in locations US)	The Impact of Positron Emission Tomography with 18F-Fluciclovine on the Treatment of Biochemical Recurrence of Prostate Cancer: Results from the LOCATE Trial	Prospective, open-label, multicentre, interventional LOCATE study looking at whether [^{18}F]Fluciclovine-PET-CT testing changes clinical management in 213 men who had had curative-intent treatment but who were suspected as having recurrence based on rising PSA levels. The study was carried out across 17 US centres.	[^{18}F]Fluciclovine-PET-CT detected 1 or more sites of recurrence in 57% of men with BCR. Overall, 59% (126/213) of patients had a change in management; 78% (98/126) of these were 'major' changes. The most frequent major changes were from salvage or non-curative systematic therapy to watchful waiting (25%), from non-curative systematic therapy to salvage therapy (24%) and from salvage therapy to non-curative systemic therapy (9%). At 6 months, 63% of patients had treatment that was concurrent with post-scan plans, a clinically important difference was found for 37%
Teoh et al. (2018) [25]<br><br>NCT02578940<br><br>(7 study locations in UK)<br><br>Note that only an abstract had been published at the time of the Medtech Innovation Briefing 2019. Full results were published in June 2020 [26]	The FALCON trial: Impact of 18F-fluciclovine PET-CT on clinical management choices for men with biochemically recurrent prostate cancer.	Prospective, open-label, multicentre, interventional FALCON study involving 85 men being considered for curative-intent salvage therapy after initial radical therapy (pre-planned interim analysis of the first 85 patients). The study was carried out across 6 UK centres.	Most of the men in this pre-planned interim analysis had previously had radical prostatectomy (65.9%), with 27 men having had salvage radiotherapy (± other therapy). After [^{18}F]Fluciclovine PET-CT imaging, 61.2% of those imaged had a change in management, suggesting [^{18}F]Fluciclovine PET-CT has an effect on clinical decisions for men with a first BCR of prostate cancer after curative- intent primary therapy. Recruitment was subsequently stopped as the pre- specified cut-off for efficacy (>45 treatment changes) was met.
Bach-Gansmo et al. (2017) [27]<br><br>NCT02443571<br><br>(4 study locations in US, Italy, Norway)	Multisite Experience of the Safety, Detection Rate and Diagnostic Performance of Fluciclovine (^{18}F) Positron Emission Tomography/Computerized Tomography Imaging in the Staging of Biochemically Recurrent Prostate Cancer	Multicentre, retrospective, observational BED-001 study involving 596 patients who had [^{18}F]Fluciclovine PET-CT at 4 centres (1 in US, 1 in Italy and 2 in Norway).	At a subject level, [^{18}F]Fluciclovine PET-CT showed a detection rate of 67.7% (403/595 scans). At a regional level, positive findings were detected in the prostate/bed in 38.7% and in pelvic lymph nodes in 32.6% of scans. Metastatic involvement outside the lymph nodes was detected in 26.2% of scans. Subject level detection rate in those in the lowest quartile for baseline PSA (0.79 ng/mL or less) was 41.4%. The positive predictive value of 18F-fluciclovine PET-CT for all sampled lesions was 62.2%, and was 92.3% and 71.8% for extraprostatic and prostate/bed involvement respectively. Treatment emergent adverse events were experienced by 5.4% of patients but none were considered adverse reactions to [^{18}F]fluciclovine. The safety profile was not noticeably altered after repeat administration.
Jani et al. (2017) [28]<br><br>NCT01666808<br><br>(1 study location in US)	Prospective, single-centre randomised, controlled clinical trial involving 96 patients with rising levels of PSA after prostatectomy being considered for salvage radiotherapy.	Prospective, single-centre randomised, controlled clinical trial involving 96 patients with rising levels of PSA after prostatectomy being considered for salvage radiotherapy	With the exception of PTV2, all post-registration volumes were statistically significantly larger than the corresponding pre-registration volumes, suggesting that, in most cases, including information from [^{18}F]Fluciclovine PET-CT in the treatment planning process may lead to larger volumes targeted for salvage therapy. This was associated with higher doses of radiotherapy (40 Gy and 60 Gy) to the penile bulb (p=0.001 and p=0.002 respectively), but no statistically significant difference in rectal or bladder doses. Acute toxicity results were not statistically significantly different between the control and experimental arms and no acute grade 3, 4 or 5

			toxicity was seen in the experimental arm, suggesting treatment to the modified clinical target is tolerable
Akin-Akintayo et al. (2017) [29]<br><br>NCT01666808<br><br>(1 study location in US)	Change in Salvage Radiotherapy Management Based on Guidance With FACBC (Fluciclovine) PET-CT in Postprostatectomy Recurrent Prostate Cancer	Randomised, prospective, intention-to-treat clinical trial involving men with PSA failure after radical prostatectomy. 87 men were recruited and 44 were randomised to the intervention.	2 patients dropped out before PET-CT scanning. 81.0% (34/42) of patients had a positive results on [^{18}F]Fluciclovine PET-CT. All 42 patients who had [^{18}F]Fluciclovine PET-CT were initially planned for radiotherapy. After [^{18}F]Fluciclovine PET-CT findings, radiotherapy decisions were changed in 40.5% patients (17/42). 4.8% (2/42) of patients had radiotherapy decisions withdrawn because of evidence of extra-pelvic disease. Of the remaining patients, 37.5% (15/40) had radiotherapy fields changed; with 73.3% (11/15) of these fields increased from prostate bed only to both prostate and pelvis, and 26.7% (4/15) reduced from both prostate bed and pelvis to the prostate bed only. Changes in radiotherapy field and overall radiotherapy decision were both statistically significant ($p<0.001$) with [^{18}F]Fluciclovine PET-CT, but change in the decision to offer radiotherapy or not was not statistically significant ($p=0.15$).
Ren et al. (2016) [29]	The value of anti-1-amino-3-18F-fluorocyclobutane-1-carboxylic acid PET-CT in the diagnosis of recurrent prostate carcinoma: a meta-analysis	Systematic review and meta-analysis of published data about the performance of [^{18}F]Fluciclovine PET-CT in the diagnosis of recurrent prostate cancer.<br>6 studies were included, involving a total of 251 patients with suspected prostate cancer recurrence.	[^{18}F]Fluciclovine PET-CT had an 87% pooled sensitivity, and a 66% pooled specificity. On a per-patient-based analysis in detecting prostate cancer recurrence, [^{18}F]Fluciclovine PET-CT had an area under the receiver-operating characteristic curve of 0.93.

Table 19: Oncology indications for [18F]FDG from the ICSCNM 2013[30] and 2016[31] reports and the commissioning status from the most recent policy in NHS England[32], NHS Scotland[33], or NHS Wales[3].

ICSCNM Report	Indication	Tracer	Commissioning Status		
**2013 or 2016**	**Oncology**	**[18F]FDG**	**NHS England**	**NHS Scotland**	**NHS Wales**
			Date of commissioning policy		
2013	Brain	[18F]FDG	2015	Not routinely commissioned	Not discussed
2013	Head and Neck	[18F]FDG	2015	2017	2015, 2018, 2020
2013	Thyroid Carcinoma	[18F]FDG	2015	2017	2018
2013	Lung Carcinoma	[18F]FDG	2015	2017	2015
2013	Pleural Malignancy	[18F]FDG	2015	Not routinely commissioned	Not discussed
2013	Thymic tumours	[18F]FDG	2015	Not routinely commissioned	Not discussed
2013	Oesophago-gastric carcinoma	[18F]FDG	2015	2017	2015, 2020
2013	Gastrointestinal stromal tumours	[18F]FDG	2015	Not routinely commissioned	Not discussed
2013	Breast Carcinoma	[18F]FDG	2015	2017	2018
2013	Hepato-pancreatico-biliary cancers	[18F]FDG	2015	Not routinely commissioned	2019
2013	Colorectal carcinoma	[18F]FDG	2015	2017	2015
2013	Urological malignancy	[18F]FDG	2015	Not routinely commissioned	2019
2013	Gynaecological malignancy	[18F]FDG	2015	2017	2015, 2019, 2020
2013	Testicular malignancy	[18F]FDG	2015	Not routinely commissioned	2015
2013	Anal and penile carcinoma	[18F]FDG	2015	Not routinely commissioned	2015
2013	Lymphoma	[18F]FDG	2015	2017	2015
2013	Skin tumours	[18F]FDG	2015	Melanoma 2017	2015
2013	Musculoskeletal tumours	[18F]FDG	2015	Not routinely commissioned	Not discussed
2013	Paraneoplastic syndromes	[18F]FDG	2015	Not routinely commissioned	Not discussed
2013	Carcinoma of unknown primary	[18F]FDG	2015	Not routinely commissioned	2015
2013	Neuroendocrine tumours	[18F]FDG	2015	Not routinely commissioned	2015
2013	Rare tumours in children and young adults:<br>Osteoscarcoma<br>Ewing's sarcoma<br>Wilm's tumours<br>Neuroblastoma<br>Hepatoblastoma<br>Kangerhans' cell histiocytosis	[18F]FDG	2015	Not routinely commissioned	Not discussed
2016	Myeloma	[18F]FDG	No	Not routinely commissioned	2015, 2018
2016	Rhabdomyosarcoma	[18F]FDG	No	Not discussed	Not discussed
NA	Pancreatic cancer	[18F]FDG	2018 NICE guidance	Check date – unclear when updated	2019

Table 20: Non-oncology indications for [^{18}F]FDG from the ICSCNM 2013[30] and 2016[31] reports and the commissioning status from the most recent policy in NHS England[32], NHS Scotland[33], or NHS Wales[3].

**ICSCNM Report**	**Indication**	**Tracer**	**Commissioning Status**		
**2013 or 2016**	**Non-oncology**	**[^{18}F]FDG**	**NHS England**	**NHS Scotland**	**NHS Wales**
			Date of commissioning policy		
2013	Vasculitis	[^{18}F]FDG	2015	2017	2019
2013	Sarcoidosis	[^{18}F]FDG	2015	Not routinely commissioned	2019
2013	Infection Imaging	[^{18}F]FDG	2015	Not routinely commissioned	2019
2013	Pyrexia of unknown origin	[^{18}F]FDG	2015	Not routinely commissioned	2019
2013	Cardiological indications<br>-Assessment of myocardial viability	[^{18}F]FDG	2015	Not routinely commissioned	Referral to Guys and St Thomas
2013	Neurological applications<br>- Pre-surgical assessment of medically refractory complex partial seizures<br>- Evaluation of memory loss/neurological signs suggestive of dementia	[^{18}F]FDG	2015	Not routinely commissioned	Not discussed

Table 21: Oncology indications for non [^{18}F]FDG radiotracers from the ICSCNM 2013[30] and 2016[31] reports and the commissioning status from the most recent policy in NHS England[32], NHS Scotland[33], or NHS Wales[3]. *An Interim Clinical Commissioning Policy Statement in 2019 commissioned [^{68}Ga]Ga-PSMA and [^{18}F]F-PSMA for a limited period of time to ensure the stability of service provision for prostate cancer patients due to pressure on production and supply of [^{18}F]Fluoroethylcholine (FEC) and [^{18}F]Fluoromethylcholine (FMC).

**ICSCNM Report**	**Indication**	**Tracer**	**Commissioning Status**		
**2013 or 2016**	**Oncology**		**NHS England**	**NHS Scotland**	**NHS Wales**
			Date of commissioning policy		
2013	Gliomas	[^{11}C]Methionine	2015	2017 - to distinguish between tumour progression and treatment effect	Not discussed
2016	Gliomas	[^{18}F]Flouroethyl-tyrosine ([^{18}F]FET)	No	Not discussed	Not discussed
2013	Parathyroid tumour	[^{11}C]Methionine	2015	Not discussed	Not discussed
	Parathyroid tumour	[^{18}F]Fluorocholine	No	Not discussed	2020
2013	Prostate cancer	[^{11}C]Choline	2015	2017 Exceptional cases	Not discussed
2013	Prostate cancer	[^{18}F]Fluoroethyl-choline (FEC)	2015	2017 Exceptional cases	2018
2013	Prostate cancer	[^{18}F]Fluoromethyl-choline (FMC)	2015	2017 Exceptional cases	2018
2016	Prostate cancer	[^{68}Ga]Ga-PSMA	2019 Interim commissioning*	2020 Routinely commissioned	2018
NA	Prostate cancer	[^{18}F]F-PSMA	2019 Interim commissioning*	Not discussed	2018
2013	Hepatocellular carcinoma	[^{11}C]Choline	2015	Not discussed	Not discussed
2013	Hepatocellular carcinoma	[^{18}F]Fluoroethyl-choline (FEC)	2015	Not discussed	Not discussed
2013	Hepatocellular carcinoma	[^{18}F]Fluoromethyl-choline (FMC)	2015	Not discussed	Not discussed
2013	Hepatocellular carcinoma	[^{11}C]Acetate	2015	Not discussed	Not discussed
2013	Neuroendocrine tumours	[^{68}Ga]Ga-SSR	2015	2017 Exceptional cases	2015
2013	Neuroendocrine tumours	[^{18}F]F-DOPA	2015	Not discussed	Not discussed
2013	Malignant diseases of bone	[^{18}F]NaF	2015	Not discussed	Not discussed

Table 22: Non-oncology indications for non [18F]FDG radiotracers from the ICSCNM 2013[30] and 2016[31] reports and the commissioning status from the most recent policy in NHS England[32], NHS Scotland[33], or NHS Wales[3].

ICSCNM Report	Indication	Tracer	Commissioning Status		
2013 or 2016	Non-oncology		NHS England	NHS Scotland	NHS Wales
			Date of commissioning policy		
2013	Cardiological indications -Assessment of myocardial viability	[^{82}Rb] Rubidium Chloride	No – except two centres in Manchester and London 2015	Not discussed	Not discussed
2013	Cardiological indications -Assessment of myocardial viability	[^{13}N]NH_3	No	Not discussed	Not discussed
2016	Cardiac Sarcardosis	[^{82}Rb]Rubidium Chloride	No	Not discussed	Not discussed
2016	Cardiac Sarcardosis	[^{13}N]NH_3	No	Not discussed	Not discussed
2013	Perfusion in patients with coronary anomalies with Kawasaki's disease.	[^{82}Rb]Rubidium Chloride	No	Not discussed	Not discussed
2013	Perfusion in patients with coronary anomalies with Kawasaki's disease.	[^{13}N]NH_3	No	Not discussed	Not discussed
2013	Hyperinsulinism	[^{18}F]F-DOPA	No	Not discussed	Not discussed
2013	Cognitive impairment – possible Alzheimers disease	[^{18}F]Florbetapir	No	No	Not discussed
2016	Cognitive impairment – possible Alzheimers disease	[^{18}F]Florbetaben	No	No	Not discussed
2016	Cognitive impairment – possible Alzheimers disease	[^{18}F]Flutemetamol	No	No	Not discussed

Table 23: The development and review process a policy must go through to be commissioned by NHS England Specialised Services as outlined in the NHS England Specialised Commissioning Service Development Policy (2017)[34], with further detail provided in a document outlining the methodology for producing National Clinical Policies[35].

**Proposal phase**	The inclusion of a new intervention into policy is proposed by a clinician, deemed qualified to be the Policy Clinical Lead, through a preliminary policy proposal (PPP) which is developed into the final policy through a three-stage process.
**Stage 1: Clinical build phase**	New or amended clinical commissioning policies are developed. New policies need to be underpinned by an independent clinical evidence review, which is conducted by NICE if the technology has been granted UK MA or application is ongoing. For all other policy propositions, the Clinical Panel will set out the type of independent evidence review required. In particular, the focus is on the patient benefit offered by the drug, device or intervention, and the quality of the evidence for clinical-effectiveness. NHS England's specialised services clinical panel challenges and confirms whether a proposal has a sound evidence base.
**Stage 2: Impact analysis phase**	The financial and operational impacts of moving from current pathways of care to the proposed pathways are identified in the draft policy proposition and are then subject to stakeholder testing and public consultation.
**Stage 3: Decision phase**	For cost-neutral or cost-saving propositions, the decision is based on an assessment of clinical benefit. There is a fixed budget for new specialised service commissioning and so *all* specialised service propositions that require additional funding are ranked based on their relative incremental clinical benefit and incremental cost, and those providing the best value for money will be funded up to the available budget. This ranking and commissioning process is carried out twice a year by the Specialised Services Commissioning Committee and the results are published on the NHS England website.

Figure 6: Overview of the Service Development process for new policies in Specialised Commissioning by NHS England. Reproduced from [36].

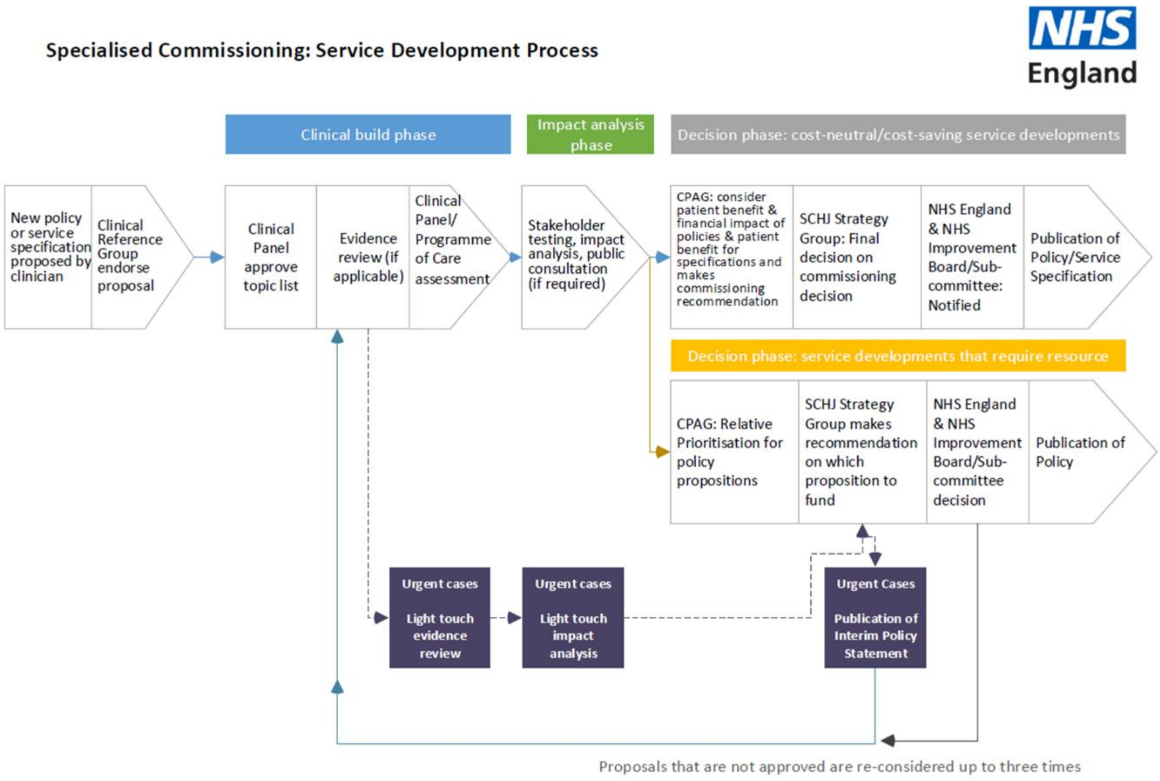

References:

1. NHS England and NHS Improvement. Diagnostic Imaging Dataset Annual Statistical Release 2019/20 Version 1.0 (Internet). (2020). Available from: https://www.england.nhs.uk/statistics/wp-content/uploads/sites/2/2020/10/Annual-Statistical-Release-2019-20-PDF-1.4MB.pdf

2. Scottish Clinical Imaging Network. PET-CT Waiting and Reporting Times (Internet). (2020). Available from: https://www.scin.scot.nhs.uk/scin-pet-ct-waiting-reporting-times/ : (accessed 2020 Dec 9)

3. Welsh Health Specialised Services Committee. Specialised Services Commissioning Policy : Positron Emission Tomography Version 6.0 (Internet). (2020). Available from: https://whssc.nhs.wales/commissioning/whssc-policies/cancer/cp50a-positron-emission-tomography-pet-pdf/

4. Medicines and Healthcare Products Regulatory Agency. Guidance Note 5. Notes for applicants and holders of a Manufacturer's Licence (Internet). Available from: https://www.gov.uk/government/publications/medicines-notes-for-applicants-and-holders-of-a-manufacturer-licence

5. Directive 2001/83/EC of the European Parliament and of the Council of 6 November 2001 on the Community code relating to medicinal products for human use (Internet). (2001). Available from: https://eur-lex.europa.eu/legal-content/EN/ALL/?uri=CELEX:32001L0083

6. Medicines and Healthcare Products Regulatory Agency. Guidance Note 14. The supply of unlicensed medicinal products ("specials") (Internet). Available from: https://www.gov.uk/government/publications/supply-unlicensed-medicinal-products-specials

7. National Institute for Health and Care Excellence. Developing NICE guidelines: the manual. Processes and methods (Internet). (2014). Available from: http://www.nice.org.uk/article/pmg20

8. NIHR Innovation Observatory. Who We Are And What We Do (Internet). (2020). Available from: http://www.io.nihr.ac.uk/what-we-do/ : (accessed 2020 Dec 16)

9. National Institute for Health and Care Excellence (NICE). Guide to the processes of technology appraisal (Internet). (2014). Available from: https://www.nice.org.uk/Media/Default/About/what-we-do/NICE-guidance/NICE-technology-appraisals/technology-appraisal-processes-guide-apr-2018.pdf

10. National Institute for Health and Care Excellence. Charging for technology appraisals and highly specialised technologies (Internet). (2020). Available from: https://www.nice.org.uk/about/what-we-do/our-programmes/nice-guidance/nice-technology-appraisal-guidance/charging : (accessed 2020 Dec 16)

11. Statutory Instruments. No. 259 NATIONAL HEALTH SERVICE, ENGLAND The National Institute for Health and Care Excellence (Constitution and Functions) and the Health and Social Care Information centre (Functions) Regulation 2013 (Internet). (2013). Available from: http://www.legislation.gov.uk/uksi/2013/259/pdfs/uksi_20130259_en.pdf

12. Department of Health and Social Care, Public Health England. Handbook to the NHS Constitution for England (Internet). (2021). Available from: https://www.gov.uk/government/uploads/system/uploads/attachment_data/file/474450/N

HS_Constitution_Handbook_v2.pdf : (accessed 2021 Jan 6)

13. National Institute for Health and Care Excellence. Guide to the methods of technology appraisal 2013 (Internet). (2018). Available from: http://doi.org/10.1183/13993003.01815-2018

14. National Institute for Health and Clinical Excellence. Diagnostics Assessment Programme Manual (Internet). Online. (2011). Available from: http://www.nice.org.uk/media/A0B/97/DAPManualFINAL.pdf

15. National Institute for Health and Clinical Excellence, Center for Health Technology Evaluation, Medical Technologies Evaluation Programme. NICE Medtech Innovation Briefings -Interim Process and Methods Statement (Internet). (2014). Available from: https://www.nice.org.uk/Media/Default/About/what-we-do/NICE-advice/Medtech-innovation-briefings/MIB-interim-process-methods-statement.pdf

16. The National Institute for Health and Care Excellence. Evidence summaries: process guide. (2017). Available from: https://www.nice.org.uk/process/pmg31/chapter/introduction

17. National Institute for Health and Care Excellence. Medicines optimisation: key therapeutic topics – Integrated process statement (Internet). (2018). Available from: https://www.nice.org.uk/process/pmg13/chapter/introduction

18. European Medicines Agency. Assessment report. EMEA/H/C/004197/0000 Committee for Medicinal Products for Human Use (CHMP) Axumin. International non-proprietary name: fluciclovine (18f). (Internet). (2017). Available from: http://www.ema.europa.eu/docs/en_GB/document_library/EPAR_-_Public_assessment_report/human/004197/WC500230836.pdf. Updated Mar 23, 2017. Accessed Jun 25, 2018.

19. European Medicines Agency. Assessment report. EMA/506460/2017 Committee for Medicinal Products for Human Use (CHMP) Lutathera. International non-proprietary name: lutetium (177lu) oxodotreotide (Internet). Vol. 44. (2017). Available from: www.ema.europa.eu/contact

20. Brabander, T., Van Der Zwan, W.A., Teunissen, J.J.M., Kam, B.L.R., Feelders, R.A., De Herder, W.W., et al. Long-term efficacy, survival, and safety of [177Lu-DOTA0,Tyr3]octreotate in patients with gastroenteropancreatic and bronchial neuroendocrine tumors. *Clin Cancer Res*. **23**(16):4617–24 (2017).

21. Strosberg, J., El-Haddad, G., Wolin, E., Hendifar, A., Yao, J., Chasen, B., et al. Phase 3 Trial of 177 Lu-Dotatate for Midgut Neuroendocrine Tumors . *N Engl J Med*. **376**(2):125–35 (2017).

22. National Institute for Health and Care Excellence. NICE Guidance. Lutetium (177Lu) oxodotreotide for treating unresectable or metastatic neuroendocrine tumours. Technology appraisal guidance (Internet). (2018). Available from: https://www.nice.org.uk/guidance/ta539

23. National Institute for Health and Care Excellence. NICE Advice. Axumin for functional imaging of prostate cancer recurrence. Medtech innovation briefing. (2019). Available from: https://www.nice.org.uk/advice/mib172

24. Andriole, G.L., Kostakoglu, L., Chau, A., Duan, F., Mahmood, U., Mankoff, D.A., et al. The Impact of Positron Emission Tomography with 18F-Fluciclovine on the Treatment of Biochemical Recurrence of Prostate Cancer: Results from the LOCATE Trial. *J Urol*.

201(2):322–30 (2019).

25. Teoh, E.J., Bottomley, D.M., Scarsbrook, A., Payne, H., Afaq, A., Bomanji, J., et al. The FALCON trial: Impact of 18F-fluciclovine PET/CT on clinical management choices for men with biochemically recurrent prostate cancer. *J Clin Oncol*. **36**(6_suppl):165–165 (2018).

26. Scarsbrook, A.F., Bottomley, D., Teoh, E.J., Bradley, K.M., Payne, H., Afaq, A., et al. Effect of 18F-Fluciclovine Positron Emission Tomography on the Management of Patients With Recurrence of Prostate Cancer: Results From the FALCON Trial. *Int J Radiat Oncol Biol Phys* (Internet). **107**(2):316–24 (2020). Available from: https://doi.org/10.1016/j.ijrobp.2020.01.050

27. Bach-gansmo, T., Nanni, C., Nieh, P.T., Zanoni, L., Bogsrud, T.V., Sletten, H., et al. Multisite Experience of the Safety, Detection Rate and Diagnostic Performance of Fluciclovine (18F) Positron Emission Tomography/Computerized Tomography Imaging in the Staging of Biochemically Recurrent Prostate Cancer. *J Urol*. **197**:676–83 (2017).

28. Jani, A.B., Schreibmann, E., Rossi, P.J., Shelton, J., Godette, K., Nieh, P., et al. Impact of 18F-fluciclovine PET on target volume definition for postprostatectomy salvage radiotherapy: Initial findings from a randomized trial. *J Nucl Med*. **58**(3):412–8 (2017).

29. Akin-akintayo, O.O., Jani, A.B., Odewole, O., Tade, F.I., Nieh, P.T., Master, V.A., et al. Change in salvage radiotherapy management based on guidance with FACBC (fluciclovine) PET-CT in postprostatectomy recurrent prostate cancer. *Clin Nucl Med*. **42**(1):1–17 (2018).

30. Intercollegiate Standing Committee on Nuclear Medicine. Evidence-based indications for the use of PET-CT in the UK 2013. (2013).

31. Inter-Collegiate Standing Committee on Nuclear Medicine. Evidence-based indications for the use of PET-CT in the United Kingdom 2016 (Internet). (2016). Available from: https://www.rcr.ac.uk/publication/evidence-based-indications-use-pet-ct-united-kingdom-2016.

32. NHS England. NHS Commissioning Board Clinical Commissioning Policy Statement : Positron Emission Tomography Computed Tomography (PET-CT) Guidelines (all ages) (Internet). (2015). Available from: https://www.england.nhs.uk/wp-content/uploads/2018/08/Positron-emission-tomography-Computed-tomography-guidelines-all-ages.pdf

33. Scottish Clinical Imaging Network. National Services Division PET-CT Review of Indications Report (Internet). (2017). Available from: https://www.scin.scot.nhs.uk/wp-content/uploads/2017/08/PET-CT-Review-of-Indications-2016-Report-V2-1.pdf

34. NHS England. NHS England Specialised Commissioning Service Development Policy (Internet). (2017). Available from: https://www.england.nhs.uk/publication/specialised-commissioning-service-development-policy-and-process/

35. NHS England and NHS Improvement. Methods: National Clinical Policies (Internet). (2020). Available from: https://www.england.nhs.uk/publication/methods-national-clinical-policies/

36. NHS England. Specialised Commissioning: Service Development process. Infographic. (2021) Available from: https://www.england.nhs.uk/publication/infographic-specialised-commissioning-service-development-process/
